# The *OsZHD1* and *OsZHD2*, Two Zinc Finger Homeobox Transcription Factor, Redundantly Control Grain Size by Influencing Cell Proliferation in Rice

**DOI:** 10.1186/s12284-025-00774-8

**Published:** 2025-03-22

**Authors:** Mingliang Guo, Chun Zheng, Chao Shi, Xiaozhuan Lu, Zeyuan She, Shuyu Jiang, Dagang Tian, Yuan Qin

**Affiliations:** 1 State Key Laboratory for Conservation and Utilization of Subtropical Agro-Bioresources, Guangxi Key Lab of Sugarcane Biology, College of Agriculture, Guangxi University, Nanning, 530004 China; 2 Biotechnology Research Institute, Fujian Provincial Key Laboratory of Genetic Engineering for Agriculture, Fujian Academy of Agricultural Sciences, Fuzhou, 350003 China; 3 State Key Laboratory of Ecological Pest Control for Fujian and Taiwan Crops, College of Life Sciences, Fujian Provincial Key Laboratory of Haixia Applied Plant Systems Biology, Haixia Institute of Science and Technology, Fujian Agriculture and Forestry University, Fuzhou, Fujian 350002 China

**Keywords:** Rice, Zinc finger-homeodomain, *OsZHD1*, *OsZHD2*, Grain size, Cell proliferation

## Abstract

Grain size is vital determinant for grain yield and quality, which specified by its three-dimensional structure of seeds (length, width and thickness). The ZINC FINGER-HOMEODOMAIN (ZHD) proteins play critical roles in plant growth and development. However, the information regarding the function in reproductive development of ZHD proteins is scarce. Here, we deeply characterized the phenotype of *oszhd1*, *oszhd2*, and *oszhd1oszhd2*. The single mutants of *OsZHD1/2* were similar with wild type. Nevertheless, the double mutant displayed dwarfism and smaller reproductive organs, and shorter, narrower, and thinner grain size. *oszhd1oszhd2* revealed a significant decrease in total cell length and number, and single cell width in outer parenchyma; reducing the average width of longitudinal epidermal cells, but the length were increased in outer and inner glumes of *oszhd1oszhd2* compared with wild-type, *oszhd1-1*, *oszhd2-1*, respectively. *OsZHD1* and *OsZHD2* encoded the nucleus protein and were distributed predominately in stem and the developing spikelets, asserting their roles in grain size. Meanwhile, yeast two-hybrid, bimolecular fluorescence complementation, and Co-immunoprecipitation assay clarified that OsZHD1 could directly interacted with OsZHD2. The differential expression analysis showed that 839 DEGs, which were down-regulated in *oszhd1oszhd2* than wild type and single mutants, were mainly enriched in secondary metabolite biosynthetic, integral component of membrane, and transporter activity pathway. Moreover, it is reliable that the altered expression of cell cycle and expansion-related and grain size-related genes were observed in RNA-seq data, highly consistent with the qRT-PCR results. Altogether, our results suggest that *OsZHD1*/2 are functional redundancy and involved in regulating grain size by influencing cell proliferation in rice.

**Supplementary Information:**

The online version contains supplementary material available at 10.1186/s12284-025-00774-8.

## Introduction

Plant development involves a systematically genetic regulatory network through encoding various proteins, which is finely tuned by a huge number of transcription factors (TF) (Kaufmann and Airoldi 2018). Among these TFs, which are regulated at different levels, play important and unique roles from gene expression to protein activity, to ensure the accomplishment of developmental stages or in response to environmental stress tolerance (Khan et al. 2018; Glazebrook 2001; Singh et al. 2002). The TF genetic regulatory network is complexity, which is linked not only to the huge number with more than 2000 in some species, but also to their diversity with a up to 50 various families based on the nature of DNA binding domains in plants (De Mendoza et al. 2013; Franco-Zorrilla et al. 2014; Lehti-Shiu et al. 2016).

The homeodomain (HD) is a conserved DNA-binding domains (BD), which contains 60-amino acid motif encoded by a homeobox (HB) gene present in transcription factors in the eukaryotic organisms (Hu et al. 2008). Zinc finger motif was found in numerous regulatory proteins (Krishna et al. 2003). Typical zinc finger harmonizes a single zinc ion to stabilize the motif as a finger-shaped loop with two pairs of conserved cysteine and/or histidine residues (Wang et al. 2015a, b). One of the HD-containing protein families, zinc finger-homeodomain (ZF-HD) proteins, whose zinc finger motif is associated to the homeodomain, were first description from the C_4_ plant *Flaveria trinervia* as potential regulators of the C_4_*PHOSPHOENOLPYRUVATE CARBOXYLASE* gene (PEPCase) (Bollier et al. 2022; Windhövel et al. 2001).

The zinc finger-homeodomain (ZHD) subfamily of homeobox genes were identified in several species: for example, 17 genes were found in *Arabidopsis*, 14 genes in rice (Hu et al. 2008), 18 in grape (Wang et al. 2014), 31 in Chinese cabbage (Wang et al. 2015a, b), 37 in wheat (Liu et al. 2021), 22 in tomato (Khatun et al. 2017), 54 in Soybeans (Park et al. 2007), 37 in cotton (Abdullah et al. 2018), 11 in chilli (Islam et al. 2023), and 13 in Cucumber (Gao et al. 2024). This diversity in gene number could be the results of genome duplication in these species and there may be functional redundancy between *ZHD* genes, thus causing difficult their functional analysis.

In recent years, the functions of ZHD members have been reported from different species. For example, soybean *GmZF-HD1* and *GmZF-HD2* bind to the promoter region of *GmCaM4*, and could be induced by pathogen inoculation (Park et al. 2007). The expression of *AtZHD1* could be inducible under salt stress, abscisic acid (ABA) treatment, and dehydration, and it specifically binds to the *ERD1* (*EARLY RESPONSE TO DEHYDRATION*) gene promoter. In addition, AtZHD1 can interact with NAM/ATAF1,2/CUC2 (NAC) proteins to improve drought stress tolerance (Tran et al. 2007). *ATHB25/ZFHD2/ZHD1* positively regulated the *GA3ox2* (GIBBERELLIC ACID3-OXIDASE 2), thus influencing GA biosynthesis, which determined seed longevity (Bueso et al. 2014). *SlZHD17* directly regulated the the genes involved in chlorophyll metabolism, chloroplast development and carotenoid biosynthesis, and it could interact with SlMYB72, SlARF4, SlBEL11, and SlTAGL1 proteins to participate in the fruit development and ripening in tomato (Shi et al. 2021).

Among the *ZHD* genes in rice, overexpression of *OsZHD1* and *OsZHD2* induces leaf curling by influencing the number and arrangement of bulliform cells (Xu et al. 2014; Yoon et al. 2020). In addition, overexpression of *OsZHD2* increases the biosynthesis of ethylene and subsequently auxin, which stimulates root growth (Yoon et al. 2020). In this study, we deeply characterized the phenotype of *oszhd1*, *oszhd2*, and *oszhd1oszhd2*. The double mutant displayed dwarfism and smaller reproductive organs, and shorter, narrower, and thinner grain size. Histological analysis indicated that the number of outer parenchymal cells was decreased in *oszhd1*, *oszhd2*, and *oszhd1oszhd2*, and the cellular shape of outer/inner glum became abnormal. Moreover, we found that the protein of OsZHD1 could interact with OsZHD2. In conclusion, these results indicate that *OsZHD1* cooperates with *OsZHD2* involving in grain size by influencing cell proliferation.

## Materials and Methods

### Plant Materials and Growth Condition

To investigate the functions of *OsZHD1* and *OsZHD2*, single mutants were generated using CRISPR/Cas9 gene-editing with 20-bp gRNAs targeting *OsZHD1* (5’ GGGATGGCGCCCAAGCCTCC 3’) and *OsZHD2* (5’ AAGCCGGGAGGTGGAGTCGG 3’), respectively (Ma et al. 2015; Xie et al. 2017). The double mutant was constructed by targeting the same sites in both *OsZHD1* and *OsZHD2* using the CRISPR/Cas9 system (Ma et al. 2015; Xie et al. 2017). The constructs were introduced into the *Agrobacterium tumefaciens* strain EHA105 and subsequently transformed into the rice cultivar Zhonghua 11 (*Oryza sativa* L., ZH11) via *Agrobacterium*-mediated transformation. To evaluate whether a mutation was present and whether the candidate mutation site in *OsZHD1* and *OsZHD2*, we observed the target site sequence of all the transgenic plants via sequencing of the PCR products using the primers listed in the Table S1. Plants were grown in the greenhouse at 80% humidity and 22–32℃ with a 14 h/10 h (light/dark) photoperiod. The 10 cm panicles of ZH11 *oszhd1-1*, *oszhd2-1*, *and oszhd1oszhd2* were collected using tweezers and microdissection needles, and All materials contained three biological replicates and were immediately frozen in liquid nitrogen and stored at -80℃ for RNA extraction.

### RNA Isolation and qRT-PCR Analysis

Total RNA of all collected samples were extracted using Plant RNeasy Mini kit (Qiagen, Hilden, Germany). 1 µg RNA was reverse transcribed using the PrimeScript RT-PCR kit (Takara) according to the manufacture’s instruction (Cai et al. 2019). The relative expression level was detected by qRT-PCR using the Bio-Rad qRT-PCR system (Foster City, CA, USA) and SYBR Premix Ex TaqII (TaKaRa Perfect Real Time) (Guo et al. 2022), The qRT-PCR program was: 95 ℃ for 30 s; 40 cycles of annealing at 95 ℃ for 5 s and extension at 60 ℃ for 35 s; 95 ℃ for 15 s (Zhao et al. 2021a; Guo et al. 2022). The rice *OsUBQ5* gene was used as an internal control (Jain et al. 2006). To evaluate the relative expression levels of the examined genes, we used the comparative ΔΔC_T_ method (Su et al. 2017). The genes specific primers were listed in Table S1, S8, and S9.

### Histological Observation and SEM

The spikelets were fixed in FAA, which obtained 50% ethanol, 5% acetic acid, and 3.7% formaldehyde, at 4 ℃ overnight and dehydrated in a series of ethanol for observing the spikelet cell size and number. After fixing with chloroform, the samples were embedded in Paraplast Plus (Sigma). The samples were sliced into 8 μm thickness samples using an RM2245 rotary microtome (Leica). Sections were dewaxed in xylene, gradually rehydrated and dehydrated before staining with toluidine blue for light microscopy (Guo et al. 2022). Fresh materials were applied directly to the scanning electron microscope (SEM) (Ren et al. 2018; Yu et al. 2020). The cell size and number in the outer and inner parenchyma layer of the spikelet hulls were measured by Image J (https://imagej.nih.gov/ij/) (Yu et al. 2020).

### Subcellular Localization of OsZHD1 and OsZHD2

The full-length coding sequences of *OsZHD1* and *OsZHD2* were amplified from WT (ZH11) cDNA using the primers listed in Table S1. The PCR fragments were cloned into the pENTER/D-TOPO vector (Invitrogen), and pENTER/D-TOPO clones were recombined into the pGWB506 vector using LR ClonaseII enzyme (Invitrogen). The recombinant construction and 35 S::GFP (vector control) were transformed into *Agrobacterium tumefaciens* (GV3101) and infiltrated with DAPI into tobacco leaves. The fluorescence signals were observed by confocal microscope (SP8, Leica, Germany), and the excitation wavelength was 488 nm and 405 nm.

### Promoter Fusion and GUS Staining

2688 bp and 2679 bp fragments upstream of *OsZHD1* and *OsZHD2* codon sequence were respectively amplified by PCR from DNA of wild-type (ZH11) using the primers listed in Table S1. Then, the products were constructed into pENTER/D-TOPO vector (Invitrogen), and the positive clones were recombined with pGWB533 vector by LR Clonase II enzyme (Invitrogen). The wild-type (ZH11) callus were transformed using the *Agrobacterium*-mediated transformation with the *pOsZHD1*: GUS and *pOsZHD2*: GUS recombinant construction, respectively (Guo et al. 2022). The transgenic plant tissues were incubated in *β*-glucuronidase (GUS) staining buffer overnight at 37 ℃ (Jefferson et al. 1987) and dehydrated in 70% ethanol to remove the chlorophyll (Jiang et al. 2012). The images were obtained using Leica (M205 FA) microscope.

### Yeast Two-Hybrid Assay

The Y2H assay was performed as previously described (Zhao et al. 2015). The full-length coding sequences of *OsZHD1* and *OsZHD2* were respectively amplified and inserted into pGADT7 (AD) and pGBKT7 (BD), which were subsequently co-transformed into the yeast strain AH109 and then grown on SD/-Trp-Leu medium. The positive clones were selected on SD/-Trp-Leu-His-Ade + X-α-gal. The primers used for this assay are listed in Table S3.

### Bimolecular Fluorescence Complementation Assay

The BiFC assay was performed as previously described (Liu et al. 2017). The full-length coding sequence of *OsZHD1* was ligated into the C-terminal fragment of yellow fluorescent protein (YFP) in pCAMBIA1300-SPYCE vector to generate the OsZHD1-cYFP construct, and the full-length coding sequence of *OsZHD2* was ligated into the N-terminal fragment of YFP in pCAMBIA1300-SPYNE vector to generate the OsZHD2-nYFP construct. These constructs were co-infiltrated into *N.benthamiana* leaves using the *Agrobacterium*-mediated transformation (Waadt and Kudla 2008). After 36 to 48 h, leaves were collected for fluorescence signal capture using a confocal microscope (SP8, Leica, Germany)(Zhou et al. 2023). The primers used for this assay are listed in Table S3.

### Co-Immunoprecipitation Assay

The Co-IP assay was performed as previously described (Zhou et al. 2023). Briefly, the OsZHD1 and OsZHD2 proteins were individually fused with GFP and Myc tag and co-expressed in the *N.benthamiana* leaves, while GFP and OsZHD2-Myc protein were co-expressed as control. Then, the OsZHD1-GFP protein and GFP were enriched by incubating with the GFP agarose beads at 4 °C, 2 h and washed by PBS buffer (phosphate-buffered saline) three times (Lu et al. 2022). The input, immunoprecipitation and Co-IP proteins were separated by sodium dodecyl sulfate-polyacrylamide gel electrophoresis (SDS-PAGE) and detected by immunoblotting with GFP and Myc antibodies. The primers used for this assay are listed in Table S3.

### Transcriptome Sequencing and DEGs Identification

After extracting the RNA of the mutations panicle, the cDNA libraries for sequencing were constructed using the NEBNext Ultra RNA Library Prep Kit for Illumina (New England Biolabs) following the manufacturer’s instructions. Transcriptome sequencing was performed on the HiSeq 2500 platform using the 100-bp paired-end reads (Zhao et al. 2020). A standard protocol was used for the RNA-seq data preprocessing. Trimmomatic script with default parameter values was used to filter low quality reads and adapter sequence (Zhao et al.; Bolger et al. 2014). The differential expression analysis was performed as previously described (Zhao et al. 2021b). HISAT2, StringTie, and DESeq2 were performed to analyze the data derived from sequencing (Kim et al. 2019; Pertea et al. 2015; Varet et al. 2016). The clean reads were mapped to the rice reference genome (MSU7.0; http://rice.plantbiology.msu.edu/index.shtml) using HISAT2. Differential expression analysis was conducted using DESeq2. Taking the correction of q-value ≤ 0.05 as a threshold, genes with log2 (fold change) > 1 were considered as upregulated and log2(FC) < − 1 as significantly down-regulated genes. FPKM values calculated by R script were used to estimate gene expression. GO and KEGG enrichment of identified DEGs were performed with the online Omicshare tools (www.omicshare.com/tools).

## Results

### Phenotypic Characterization of the *OsZHD1* and *OsZHD2* Mutants

Figueiredo et al. (2012) identified that OsZHD1, OsZHD2, OsZHD4, and OsZHD8 bind to the promoter sequence of *OsDREB1b*. AtZHD1 is the closest homologue to OsZHD1 and OsZHD2, while AtZHD4 is to OsZHD4 and AtZHD8 to OsZHD8, and OsZHD2 is the most homologous to OsZHD1, with 80.8% identity and 84% similarity at the amino acid sequence level. However, OsZHD4 and OsZHD8 have 41.8% identity and 51.2% similarity, 41% identity and 50.9% similarity to OsZHD1, respectively (Fig. S1). Then we focus on OsZHD1 and OsZHD2 for this study. To study the role of *OsZHD1* and *OsZHD2* in regulating rice vegetative and reproductive development, we constructed the knockout vector with guide RNA (gRNA) and plant-optimized Cas9 driven by the rice *OsU6a* and maize *pUBI-H* promoters, respectively (Ma et al. 2015; Xie et al. 2017). After that, the vectors were introduced into calli of ZH11 rice by *Agrobacterium tumefaciens*-mediated transformation.

**Fig. 1:**
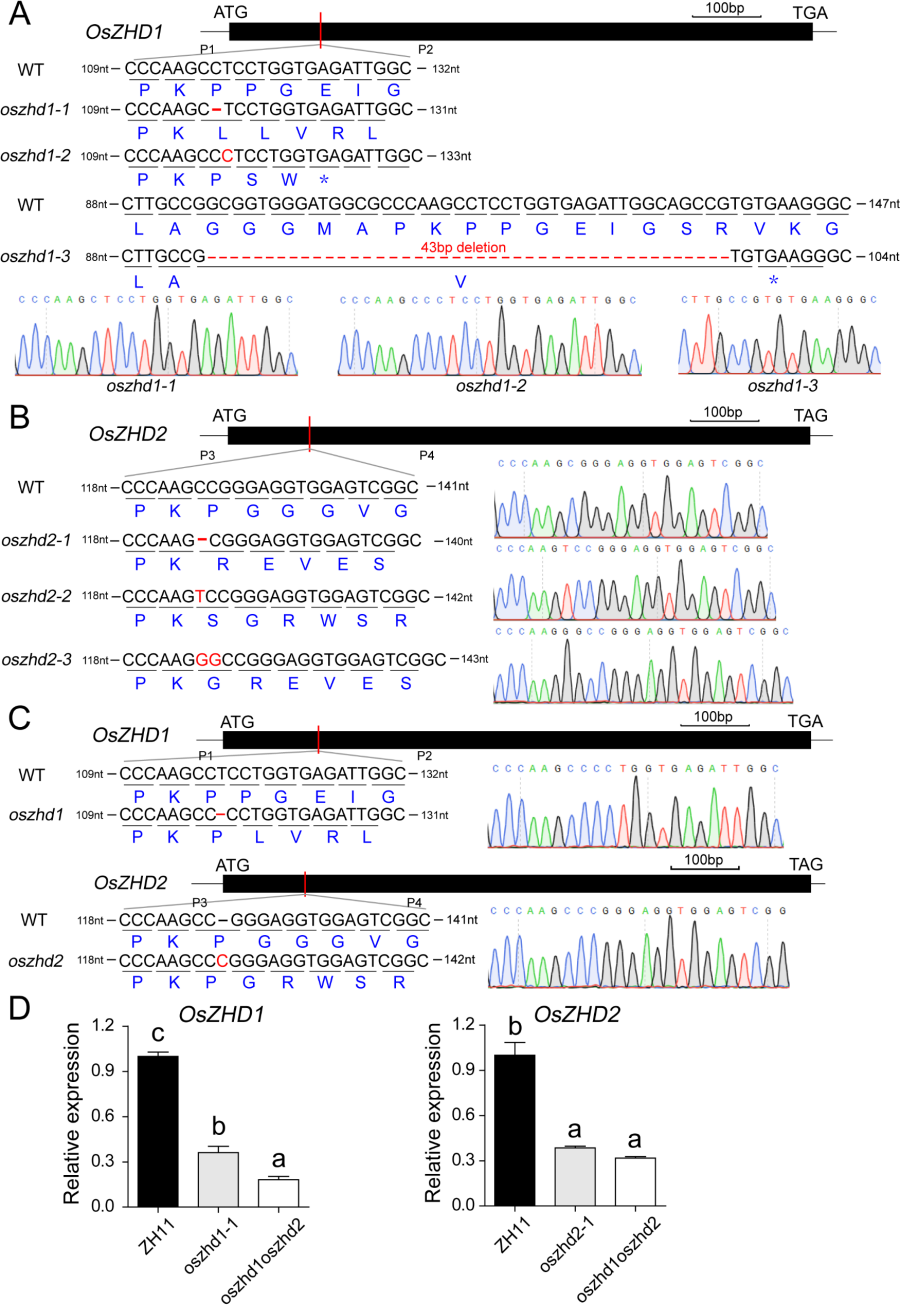
Genotyping analysis of *oszhd1*, *oszhd2*, and *oszhd1oszhd2*. (**A**) Identification of three CRISPR/Cas9-mediated mutations in *OsZHD1*. (**B**) Identification of three CRISPR/Cas9-mediated mutations in *OsZHD2*. (**C**) Identification the double mutant mediated by CRISPR/Cas9. (**D**) The expression levels of *OsZHD1* and *OsZHD2* in the mutants. The gene structure of *OsZHD1* (**A**), *OsZHD2* (**B**), and both (**C**) are respectively shown at the top. The primers of P1 and P2, P3 and P4 were used for identifying *oszhd1*, *oszhd2*, and *oszhd1oszhd2* in the T2 generation. The diagrams show the sequences of the CRISPR mutations in *OsZHD1* and *OsZHD2*. The black letters indicated the base pairs with WT in the target site. The dotted lines indicated nucleotide deletions, and dark blue letters indicated the amino acids encoded by the WT, *oszhd1*,* oszhd2*,* and oszhd1oszhd2* sequences. Data are given as means ± SD. Different letters denote significant difference at *P* < 0.05 according to ANOVA in combination with Duncan’s multiple range test

Then we generated different mutant lines (*oszhd1-1*, *oszhd1-2*, *oszhd1-3*, *oszhd2-1*, *oszhd2-2*, *oszhd2-3*, and *oszhd1oszhd2*) in the T2 generation. The *oszhd1-1* generates a frameshift mutation resulting from a 1-bp (C) deletion at position C116bp of the coding sequence (CDS). The *oszhd1-2* contains a 1-bp (C) insertion at positions C117 bp, and *oszhd1-3* contains a 43-bp deletion at positions C95-137 bp. The protein sequences of OsZHD1 were altered, and its function was knocked out in the three mutant lines (Fig. 1A). Similarly, the *oszhd2-1*, *oszhd2-2*, and *oszhd2-3* lines contained a 1-bp deletion, 1-bp insertion, and 2-bp insertion in *OsZHD2*, respectively, which led to frame-shift mutations that altered protein sequences and absence of *OsZHD2* function (Fig. 1B). Additionally, the *oszhd1oszhd2* line contained a 1-bp deletion in *OsZHD1* and a 1-bp insertion in *OsZHD2*, respectively (Fig. 1C). The qRT-PCR analysis showed that the expression of *OsZHD1* and *OsZHD2* in the CRISPR lines were significantly lower compared with the wild type (ZH11) ( Fig. 1D) using the primers of Table S1.

**Fig. 2:**
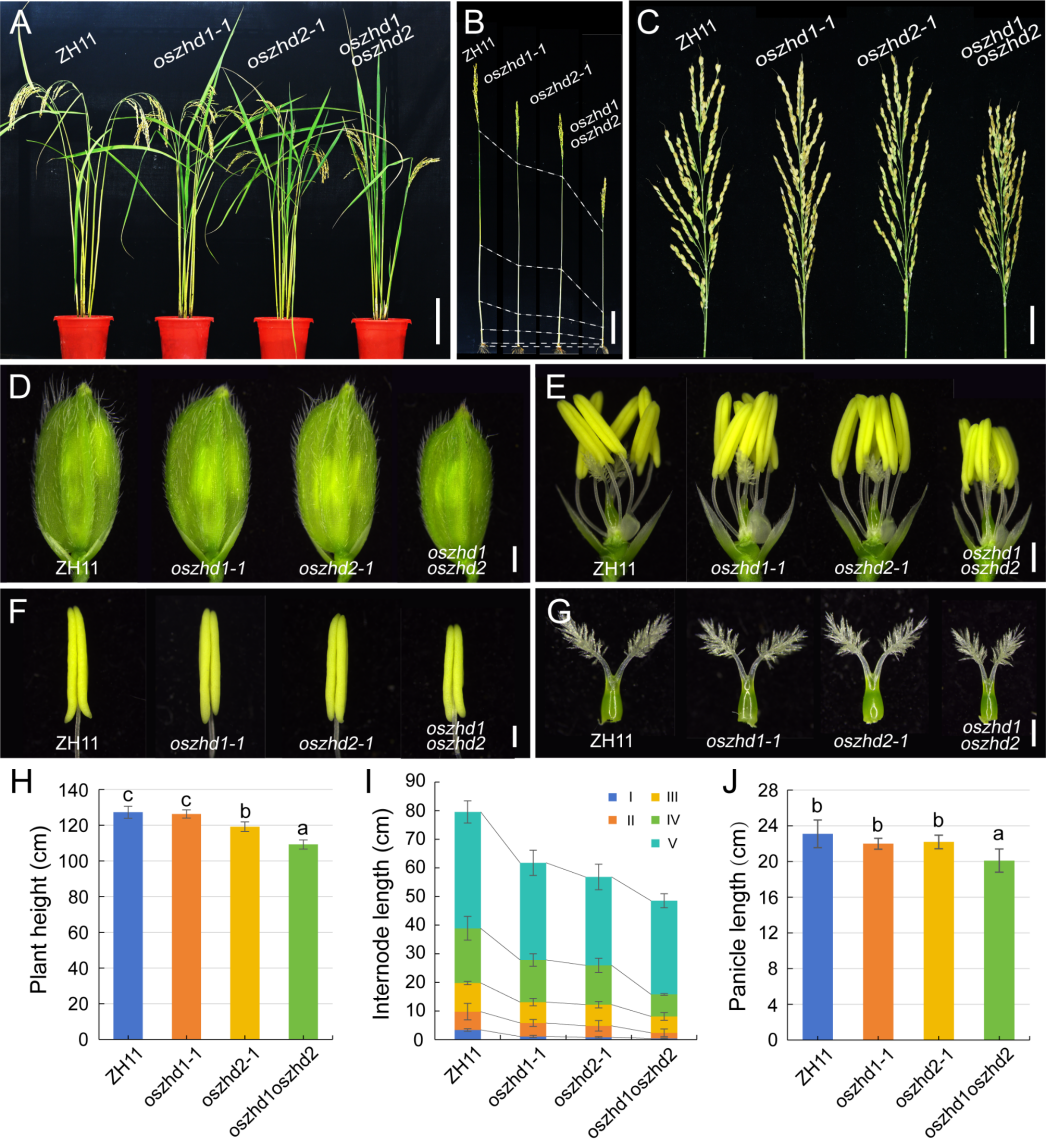
The *oszhd1oszhd2* exhibited dwarfism and smaller spikelets. (**A**) The phenotype of wild-type (left, ZH11), *oszhd1-1*, *oszhd2-1*, *and oszhd1oszhd2* plants at maturity. Bar = 15 cm. (**B-C**) The internodes length and panicle of ZH11, *oszhd1-1*, *oszhd2-1*, *and oszhd1oszhd2.* Bar = 10 cm. (**D-E**) The spikelets of ZH11, *oszhd1-1*, *oszhd2-1*, *and oszhd1oszhd2*. Bar = 1 mm. (**F-G**) Anthers and pistils of ZH11, *oszhd1-1*, *oszhd2-1*, *and oszhd1oszhd2*, respectively. Bar = 500 μm. (**H-J**) The average plant height, internode length, panicles length of ZH11, *oszhd1-1*, *oszhd2-1*, *and oszhd1oszhd2*, respectively. (*n* = 20 per sample). Data are given as means ± SD. Different letters denote significant difference at *P* < 0.05 according to ANOVA in combination with Duncan’s multiple range test

To assess the effects of disrupted *OsZHD1* and *OsZHD2* function on vegetative and reproductive growth, plant height, internode length, panicle morphology, and grain size were investigated in the three *oszhd1* and *oszhd2* lines at the mature stage, respectively. Compared to the wild type, all three mutants had no obviously reduced in the plant height, panicle, spikelet, stamen, and pistil size except internode length in *oszhd1* (Fig. S2; Table S2). Similarly, *oszhd2* three lines also had no apparently reduced in the reproductive organ tissues except plant height and internode length (Fig. S3; Table S2). However, double mutant had significantly reduced plant height (Fig. 2A, B H, and I; Table S2) and reproductive organ tissues size, such as panicle (Fig. 2C and J), spikelet (Fig. 2D and E), stamen, and pistil in *oszhd1oszhd2* compared with ZH11 and single mutants (Fig. 2F and G). This result suggesting that *OsZHD1* and *OsZHD2* involved in the regulating on plant height and reproductive organs size together.

### *OsZHD1* and *OsZHD2* Positive Influences Grain Size in Rice

**Fig. 3:**
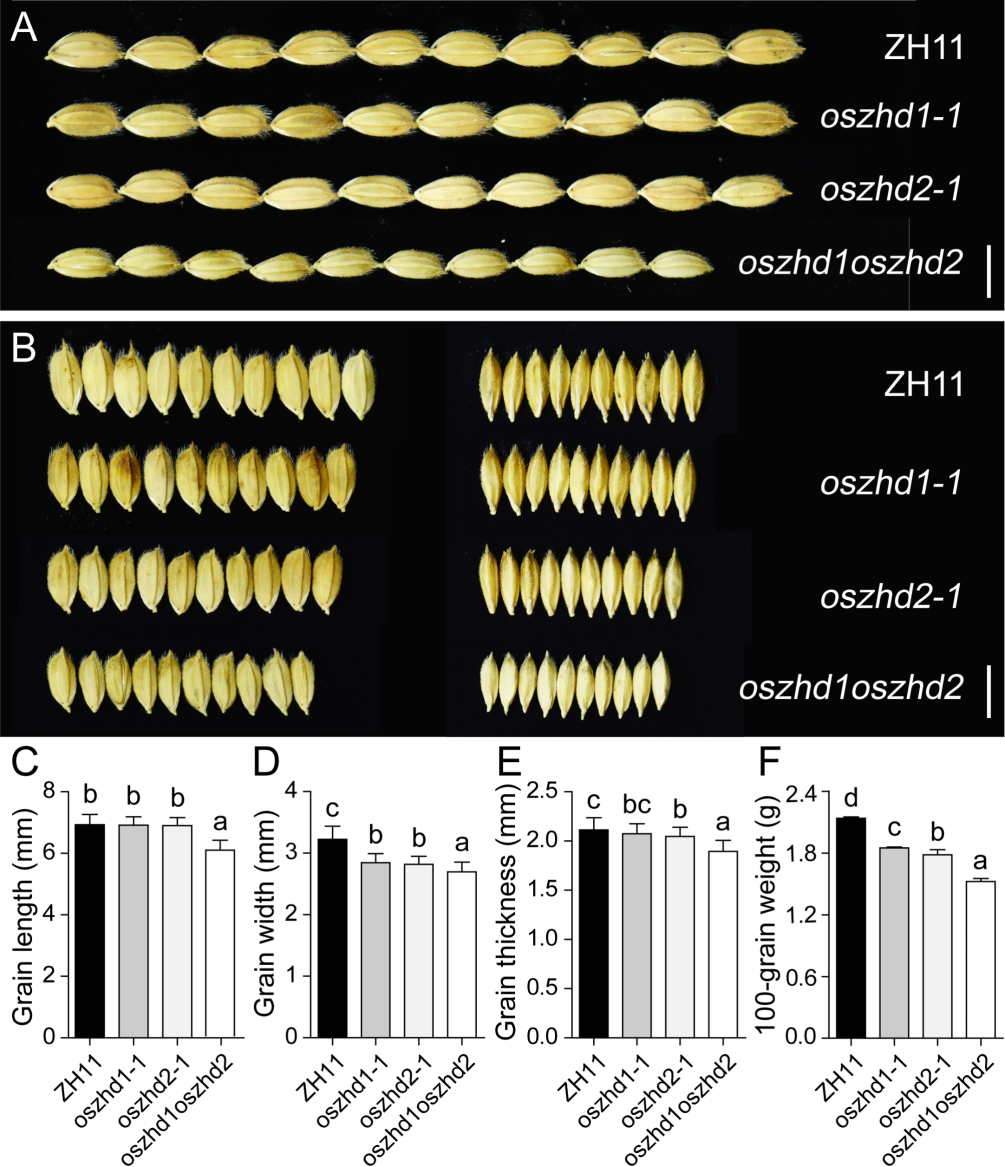
Functional verification of *OsZHD1* and *OsZHD2* in grain size. (**A-B**) Comparation on grain size of *oszhd1-1*, *oszhd2-1*, and *oszhd1oszhd2* with ZH11, respectively. Bar = 5 mm. (**C-F**) Grain length, width, thickness, and 100-grain weight of ZH11, *oszhd1-1*, *oszhd2-1*, *and oszhd1oszhd2*. (*n* = 60 per sample). Data are given as means ± SD. Different letters denote significant difference at *P* < 0.05 according to ANOVA in combination with Duncan’s multiple range test

Grain size is one of the significantly determining grain yield. To further confirm the effect of *OsZHD1* and *OsZHD2* on grain size, we examined the grain length, width, grain thickness, and 100-grain weight. The results showed that *OsZHD1* three mutant lines had significantly reduced grain width of 11.69%, 14.67%, and 16.22% than wild type in *oszhd1-1*, *oszhd1-2*, and *oszhd1-3*, respectively (Fig. 3A and B; Fig. S4A, B, and D). In addition, we found that the grains of the *OsZHD2* knockout lines were narrower and thicker than the ZH11 (Fig. S5). Three mutant lines had significantly reduced grain width of 12.61%, 14.45%, and 14.37% than wild type in *oszhd2-1*, *oszhd2-2*, and *oszhd2-3*, respectively (Fig. 3A and B; Fig. S5A, B, and D). At the same time, it reduced 3.21%, 3.78%, and 2.83% in grain thickness, respectively (Fig. 3A and B; Fig. S5A, B, and E). It is noteworthy that the grains of the double mutations with *OsZHD1* and *OsZHD2* were remarkably smaller than those of the WT, *oszhd1-1*, and *oszhd2-1* (Fig. 3). The results showed that *oszhd1oszhd2* had significantly reduced grain length of 11.43%, 11.23%, and 11.09% than the wild-type (ZH11), *oszhd1-1*, and *oszhd2-1* (Fig. 3A, B and C), 16.41%, 5.34%, and 4.35% in grain width (Fig. 3D), and it decreased 10.33%, 8.57%, and 7.35% in grain thickness (Fig. 3E). The 100-grain seed weight of the *oszhd1oszhd2* knockout line was 1.52 g, which was significantly lower than the 2.13 g, 1.85 g, and 1.78 g of the wild-type (ZH11), *oszhd1-1*, and *oszhd2-1* (Fig. 3F). These results indicated that *OsZHD1* and *OsZHD2* participate in the regulating grain size in rice.

### *OsZHD1* Cooperated With *OsZHD2* Regulating Grain Size by Affecting Cell Proliferation

**Fig. 4:**
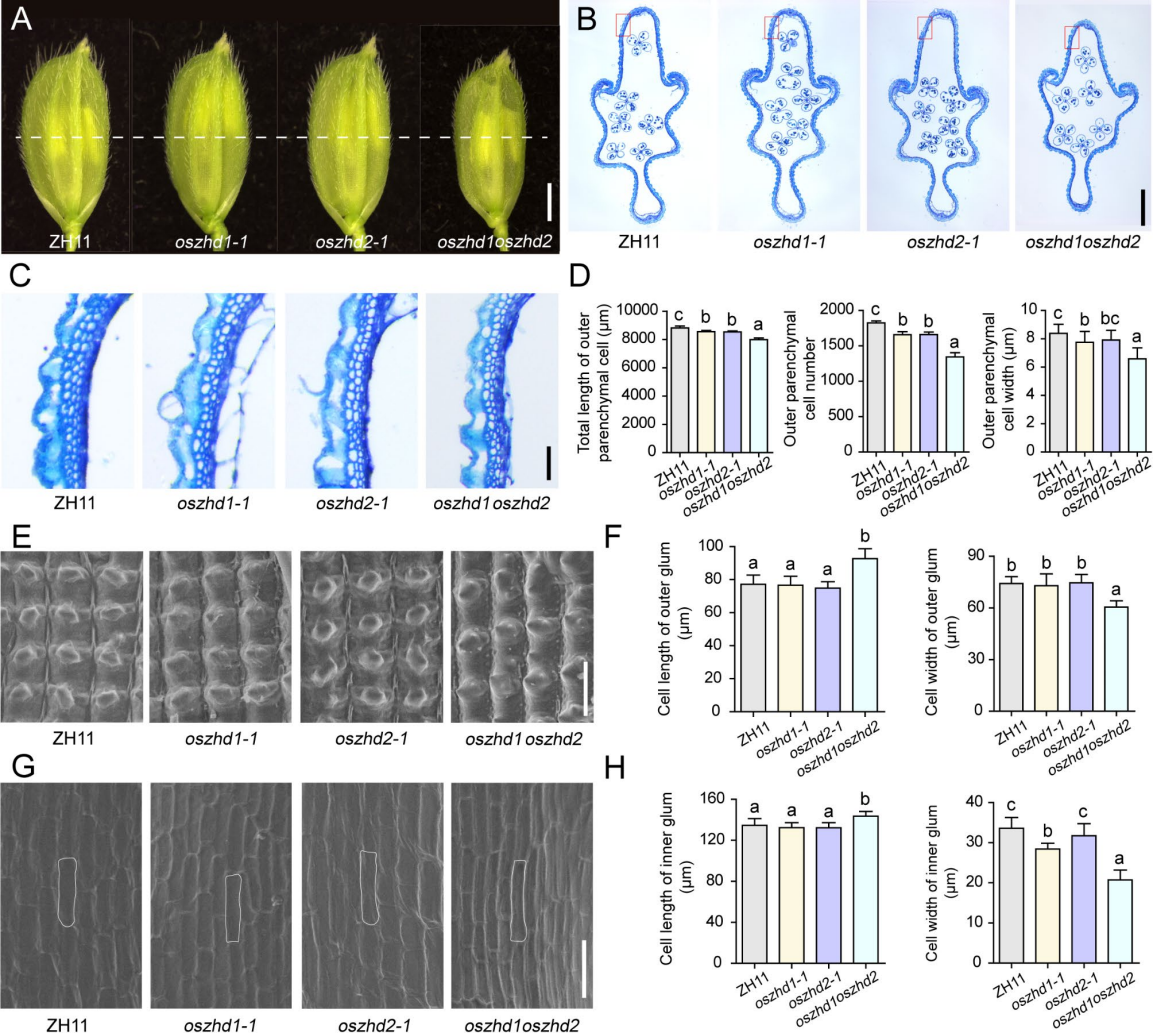
Histological analysis of spikelet hulls. (**A**) Young spikelet hulls of ZH11, *oszhd1-1*, *oszhd2-1*, *and oszhd1oszhd2*. The white line indicates the position of the cross-section. Bar = 1 mm. (**B**) Cross-sections of spikelet hulls from ZH11, *oszhd1-1*, *oszhd2-1*, *and oszhd1oszhd2*, respectively. Bar = 500 μm. (**C**) Magnified view of the cross-section area boxed in (**B**). Bar = 20 μm. (**D**) Statistical data of the total length, cell number and width in the outer parenchyma layer (*n* = 12). (**E**) Scanning electron microscope (SEM) analysis of the outer surface of glumes. Bar = 100 μm. (**F**) Statistical analysis of cell length and width in outer glumes (*n* = 40). (**G**) Scanning electron microscope (SEM) analysis of the inner surface of glumes. Bar = 100 μm. (**H**) Statistical analysis of cell length and width in inner glumes (*n* = 60). Data are given as means ± SD. Different letters denote significant difference at *P* < 0.05 according to ANOVA in combination with Duncan’s multiple range test

The spikelet hull in *oszhd1oszhd2* became smaller compared with wild type, *oszhd1-1*, and *oszhd2-1* before fertilization (Fig. 4A). Since the cell division and/or cell expansion are responsible for alteration in the final spikelet hull size and grain size, we carefully examined the cross-section of the central part of glum in mature spikelets and compared the epidermal cells of *oszhd1oszhd2* with wild-type, *oszhd1-1*, and *oszhd2-1* by scanning electron microscope. The hull cross-section of the spikelet in *oszhd1oszhd2* revealed a significant decrease of 9.37%, 6.48%, and 6.31% in total cells length (Fig. 4B, C, and D), 26.37%, 18.91%, and 19.03% in cells number of outer parenchyma, and 21.41%, 15.06%, and 16.74% in the outer parenchyma cell width compared with wild type, *oszhd1-1*, and *oszhd2-1*, respectively (Fig. 4D). The data suggested that cell division was significantly decreased in a transverse direction in the *oszhd1oszhd2* spikelet hulls.

In addition, we observed the average length and width of longitudinal epidermal cells in outer and inner glumes of wild-type, *oszhd1-1*, *oszhd2-1*, and *oszhd1oszhd2* by scanning electron microscopy. Compared with wild-type, *oszhd1-1*, and *oszhd2-1*, an apparent 20.02%, 20.91%, and 23.83% increase in cell length was observed, but 18.51%, 17.07%, and 18.91% in cell width was decreased in the outer glum, respectively (Fig. 4E and F). An apparent 6.55%, 8.47%, and 8.51% increase in cell length was observed, but 38.26%, 27.09%, and 34.66% in cell width was decreased in the inner glum, respectively (Fig. 4G and H). These observations suggest that *OsZHD1* cooperated with *OsZHD2* might suppress latitudinal growth by decreasing cell proliferation.

### OsZHD1 and OsZHD2 Localization and Expression Pattern

**Fig. 5:**
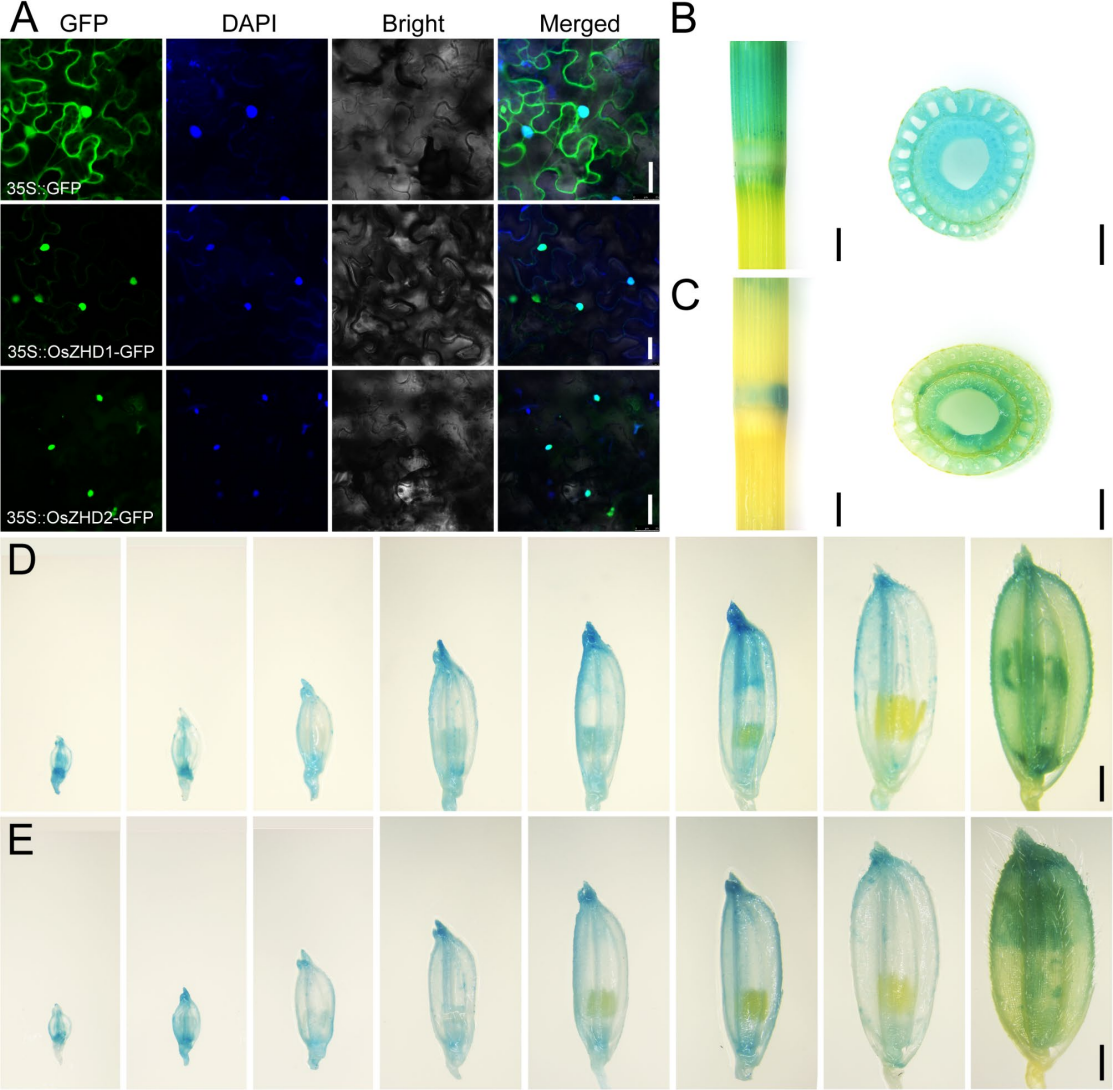
The expression profile of *OsZHD1* and *OsZHD2.* (**A**) Subcellular localization of OsZHD1 and OsZHD2 proteins were respectively shown to target the nuclear by transient expression of 35::OsZHD1-GFP and 35::OsZHD2-GFP in tobacco. The upper row is the 35 S::GFP as the control. Bar = 50 μm. (**B-E**) Expression pattern detected in transgenic plants carrying the *pOsZHD1*::GUS and *pOsZHD2*::GUS vector in ZH11 background. GUS signal was detected in the stem node of *pOsZHD1*::GUS (**B**) and *pOsZHD2*::GUS (**C**), respectively. Developing spikelets in turn 1, 2, 3, 4, 5, 6,7 mm, and mature spikelet of *pOsZHD1*::GUS (**D**) and *pOsZHD2*::GUS (**E**), respectively

To test the subcellular localization of OsZHD1 and OsZHD2, we performed a transient transformation assay. When OsZHD1/2-GFP (green fluorescent protein) fusion protein under the control of CaMV-35 S promoter were transformed into tobacco leaves, the signals mainly co-localized with the blue fluorescence of the nuclear marker protein, suggesting that OsZHD1 and OsZHD2 localizes in the nucleus (Fig. 5A).

To further examine the expression profile of *OsZHD1* and *OsZHD2*, a construct was produced in which the *OsZHD1/2* promoter (approximately 2.5 kb upstream of the ATG site) were fused to the GUS gene (*β*-glucuronidase) to transform wild-type (ZH11) plants. The results showed that OsZHD1 (Fig. 5B and D) had the similar GUS activity with OsZHD2 (Fig. 5C and E), which were detected in the internode at mature stage and the spikelets at different developmental stages. This expression profile indicated that OsZHD1 and OsZHD2 functioned in the nucleus to regulate the plant height and grain size.

### OsZHD1 Directly Interacted With OsZHD2

**Fig. 6:**
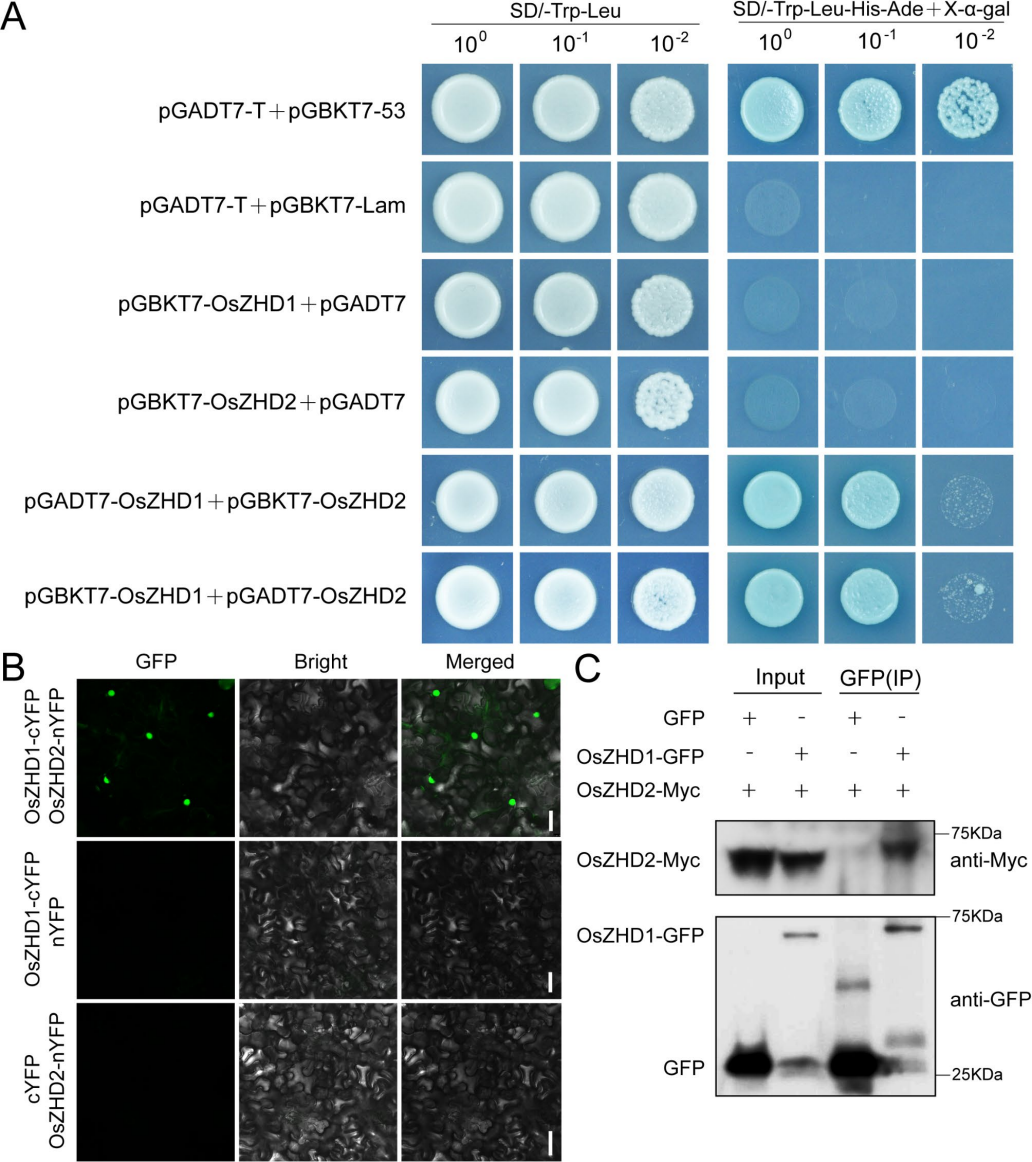
OsZHD1 interacts with OsZHD2. (**A**) The OsZHD1-OsZHD2 interaction as revealed by Y_2_H assays in yeast cells. SD, yeast dropout culture medium; PGADT7, activation domain; PGBKT7, DNA-binding domain. (**B**) BiFC analysis in *N.benthamiana* leaves of the interaction between OsZHD1-cYFP and OsZHD2-nYFP. The split YFP system was used. cYFP and nYFP are empty vectors. The YFP signals were detected with a confocal microscope. Bar = 50 μm. (**C**) In vivo Co-IP assays of OsZHD1-GFP and OsZHD2-Myc. The fusion proteins were transiently co-expressed *N.benthamiana* (tobacco) leaves. Protein extracts (input) were immunoprecipitated using an anti-GFP antibody (IP)

To determine whether OsZHD1 directly interacts with OsZHD2, we performed a yeast two-hybrid (Y2H) assay. The full length coding sequence (CDSs) of OsZHD1 and OsZHD2 were cloned and inserted into pGBKT7 (BD) and pGADT7 (AD), respectively, and co-transformed into the yeast strain AH109. The yeast containing the BD-OsZHD1 and AD-OsZHD2, or BD-OsZHD2 and AD-OsZHD1 plasmids grew well in the selection medium (Fig. 6A), while those containing BD-OsZHD1 and AD plasmids or BD-OsZHD2 and AD plasmids did not grow (Fig. 6A). This indicated that OsZHD1 and OsZHD2 interact with each other in yeast. In BiFC assay, there was no signal when OsZHD1-cYFP and nYFP, or cYFP and OsZHD2-nYFP were co-transformed into *N.benthamiana* leaves (Fig. 6B). When OsZHD1-cYFP was co-expressed with OsZHD2-nYFP in *N.benthamiana* leaves, the fluorescence signal was detected mainly in the nucleus (Fig. 6B). We also transiently expressed the OsZHD1-GFP fusion protein and the OsZHD2-Myc fusion protein in *N.benthamiana* leaves. OsZHD2-Myc was pulled down in Co-IP experiments by the beads containing OsZHD1-GFP, but not by the beads with GFP protein alone. Co-IP assays in *N. benthamiana* leaves biochemically validated the interactions (Fig. 6C). These results indicated that OsZHD1 can directly interact with OsZHD2 in the nucleus.

### Global Gene Expression Profiling in Inflorescence of the *OsZHD1* and *OsZHD2* Mutants

The spikelet hull development is vital for the final grain size. To identify potential genes involved in the grain size of *OsZHD1* and *OsZHD2* mutants, we performed transcriptome analyses using the total mRNA prepared from the inflorescences of ZH11, *oszhd1-1*, *oszhd2-1*, and *oszhd1oszhd2* at 8 cm length, respectively. Three biological replicates were conducted for these four inflorescences samples. In total, 12 samples were harvested for RNA-seq analysis in this study. The number of clean reads for the 12 samples ranged from 32.36 to 39.28 million (Table S4). For these samples, 92.45–94.68% of the clean reads were mapped to the rice genome (http://rice.plantbiology.msu.edu), with no more than two base pair mismatches in the alignment; 81.08–87.58% of the reads were uniquely mapped (Table S4). These data indicate that the quality and quantity of the reads were sufficient for quantitative gene expression analysis.

**Fig. 7:**
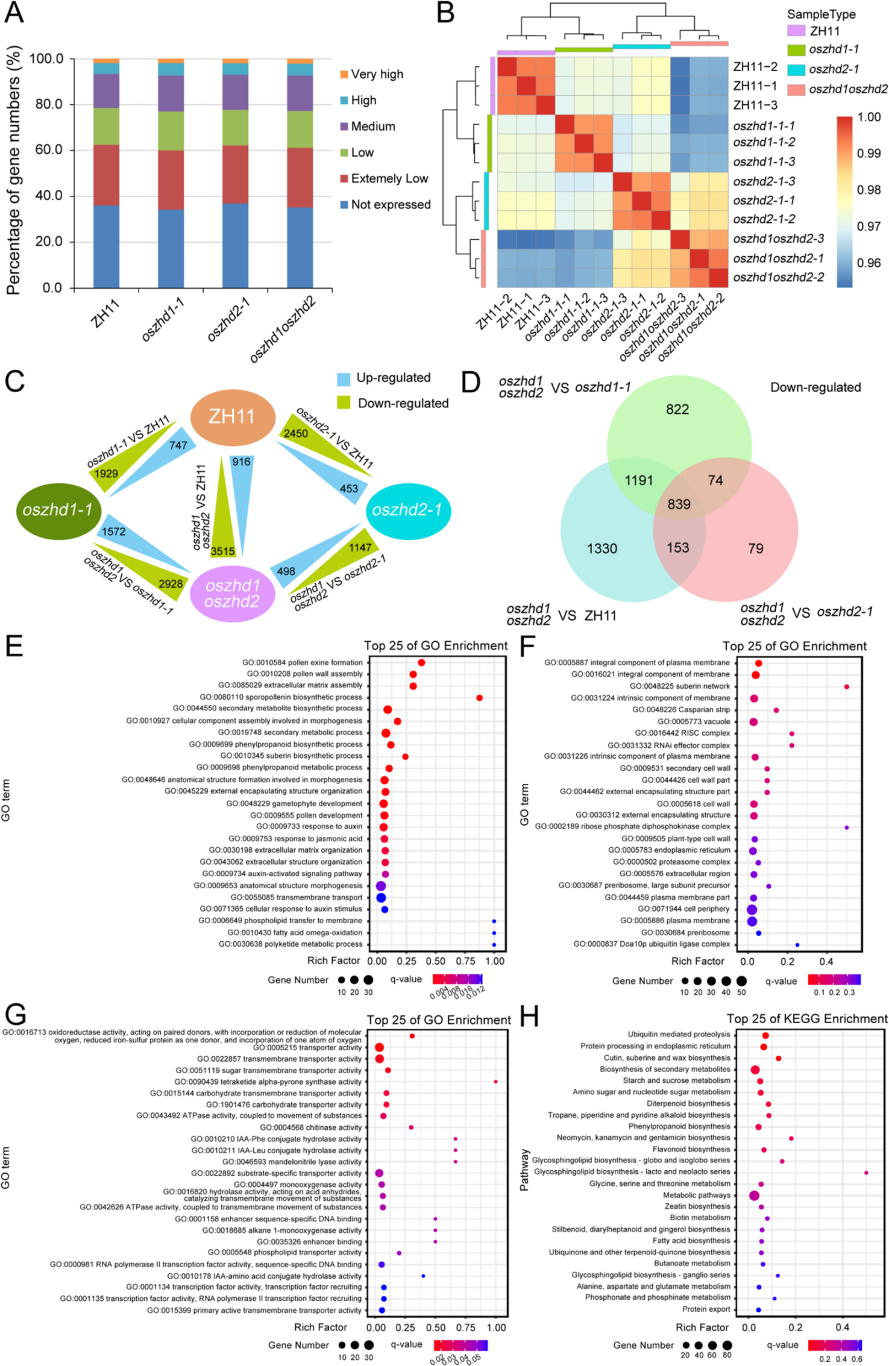
Global expression levels, DEGs, GO, and KEGG pathway analysis. (**A**) Percentage of gene numbers in each tissue according to their expression levels based on FPKM values. (**B**) Hierarchical clustering of the twelve panicle samples using Pearson correlation coefficients. (**C**) Numbers of differentially expressed genes among ZH11, *oszhd1-1*, *oszhd2-1*, *and oszhd1oszhd2* tissues. (**D**) Numbers of down-regulated genes in *oszhd1oszhd2* versus ZH11, *oszhd1-1*, and *oszhd2-1*, respectively. (**E-G**) GO classifications for all down-regulated genes in *oszhd1oszhd2* versus ZH11, *oszhd1-1*, and *oszhd2-1*. The results were classified into three main categories: biological process (**E**), cellular component (**F**), and molecular function (**G**). (**H**) KEGG pathway analysis for all down-regulated genes in *oszhd1oszhd2* versus ZH11, *oszhd1-1*, and *oszhd2-1*

For global gene expression profiling of the different samples, the FPKM (fragments per kilobase of transcript per million mapped reads) method was used to evaluate the gene expression levels by normalizing the read counts. According to their expression levels, the genes were classified into six different groups: not expressed genes, with FPKM < 1; extremely low-expression genes, with 1 ~ 10 FPKM values; low-expression genes, with 10 ~ 30 FPKM values; medium-expression genes, with 30 ~ 100 FPKM values; high-expression genes, with 100 ~ 300 FPKM values; and very high-expression genes, with FPKM values > 300. The counts of expressed genes and expression distribution pattern were highly similar among the four samples (Fig. 7A; Table S5). Hierarchical clustering of the transcriptomes from ZH11, *oszhd1-1*, *oszhd2-1*, and *oszhd1oszhd2* samples showed that the three biological replicates of each sample were grouped together with high correlation (Fig. 7B). The above results suggested the three biological replicates for each tissue good reproducibility among in this study.

### Analysis of Differentially Expressed Genes in Inflorescence of the *OsZHD1* and *OsZHD2* Mutants

In order to describe the network of gene expression that underlies *oszhd1*, *oszhd2*, and *oszhd1oszhd2*, differential expression analysis was performed using DESeq2 (Zhao et al. 2021a). Of the differential expression genes, 747 and 1929 genes were significantly up-regulated and down-regulated in *oszhd1-1* versus ZH11, respectively, and 453 and 2450 genes were significantly up-regulated and down-regulated in *oszhd2-1* versus ZH11, respectively. In addition, 916 and 3515 genes were significantly up-regulated and down-regulated in *oszhd1oszhd2* versus ZH11, respectively, 1572 and 2928 genes were significantly up-regulated and down-regulated in *oszhd1oszhd2* versus *oszhd1-1*, respectively, and 498 and 1147 genes were significantly up-regulated and down-regulated in *oszhd1oszhd2* versus *oszhd2-1*, respectively (Fig. 7C).The results in the Venn diagram showed that 839 genes were significantly down-regulated at the same time in the three comparison groups (Fig. 7D; Table S6) indicating that they may be involved in regulating spikelet/inflorescence development with *OsZHD1* and *OsZHD2*.

To further explore the expression differences in *oszhd1*, *oszhd2*, and *oszhd1oszhd2*, we investigated the biological roles of the 839 DEGs using a GO annotation analysis. GO enrichment analysis showed that these differentially expressed genes were significantly enriched in biological processes (Fig. 7E), cell components (Fig. 7F), and molecular functions (Fig. 7G). These DEGs were enriched for the GO terms secondary metabolite biosynthetic process (GO:0044550), secondary metabolic process (GO:0019748), anatomical structure formation involved in morphogenesis (GO:0048646), gametophyte development (GO:0048229), pollen development (GO:0009555), and response to auxin (GO:0009733) in biological processes (Fig. 7E). Integral component of plasma membrane (GO:0005887) and integral component of membrane (GO:0016021) were enriched in cellular components GO terms (Fig. 7F). Transporter activity (GO:0005215) and transmembrane transporter activity (GO:0022857) were enriched in molecular function GO terms (Fig. 7G).

To further investigate the role of *OsZHD1* and *OsZHD2* in grain size regulation, we conducted KEGG enrichment metabolic pathway analysis on 839 DEGs in single/double mutant lines and ZH11 in young inflorescences, and selected the top 25 metabolic pathways with the lowest q-value for mapping and analysis. The results showed that 839 DEGs were enriched in several metabolic pathways, such as ubiquitin mediated proteolysis, protein processing in endoplasmic reticulum, cutin, suberine and wax biosynthesis, and biosynthesis of secondary metabolites, and starch and sucrose metabolism (Fig. 7H). These data showed that down-regulation of *OsZHD1* and *OsZHD2* mainly affect secondary metabolite biosynthetic, integral component of membrane, and transporter activity, indicating that *OsZHD1* and *OsZHD2* play a crucial role in multiple processes of plant growth and development.

### Validation of Gene Expression Patterns by qRT-PCR

**Fig. 8:**
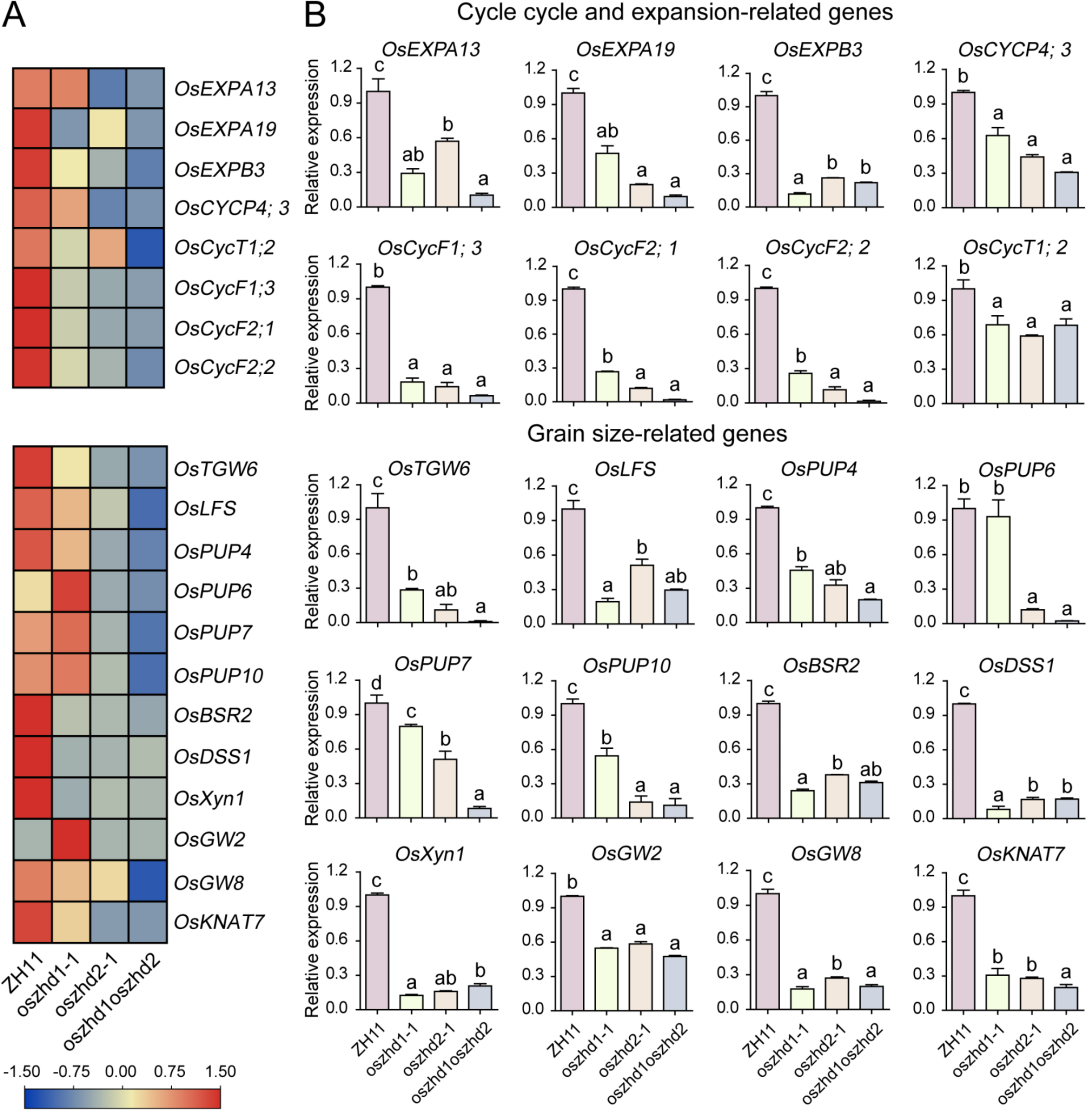
Validation of the expression patterns of cell cycle and expansion-related and grain size-related genes in the panicle of *OsZHD1* and *OsZHD2* mutants by qRT-PCR. (**A**) Expression profiling based on the FPKM values from RNA-seq analysis. (**B**) Expression patterns of genes encoding cell cycle and expansion-related and grain size-related genes by qRT-PCR. Data are given as means ± SD. Different letters denote significant difference at *P* < 0.05 according to ANOVA in combination with Duncan’s multiple range test

According to the results of histological observation and SEM, the absence of *OsZHD1* and *OsZHD2* resulted in abnormal cell quantity and morphology in the spikelet hulls, further leading to abnormal grain size. To validate the accurate of results from RNA-seq analysis, we detected related genes in inflorescences (length 8 cm) among the wild type, *oszhd1-1*, *oszhd2-1*, and *oszhd1oszhd2* using the primers listed in the Table S8 by qRT-PCR. The above results showed that cell division was significantly decreased in a transverse direction in the *oszhd1-1*, *oszhd2-1*, and *oszhd1oszhd2* spikelet hulls. Then we investigated the expression levels of genes involved in the cell expansion (*OsEXPA13*, *OsEXPA19*, *OsEXPB3*) and cell cycle (*OsCYCP4;3*, *OsCycT1;2*, *OsCycF1;3*, *OsCycF2;1*, *OsCycF2;2*) (Xu et al. 2019; Zhou et al. 2023; La et al. 2006). The RNA-seq data and qRT-PCR results showed that these genes had similar expression patterns and were down-regulated in both single and double mutants. This down-regulation might have led to the decreased cell width and number observed in the outer parenchyma (Fig. 4D). It could be attributed to the reduced expression of genes that normally suppress cell expansion and cell cycle progression, thereby affecting cell proliferation. (Fig. 8A and B; Table S7).

Grain size is vital determinant for grain yield and quality, which specified by its three-dimensional structure of seeds (length, width, and thickness). Up to now, multiple quantitative trait loci (QTL) have been cloned and researched, such as *OsTGW6*, *OsLFS*, *OsPUP4*/*BG3*, *OsPUP7*, *OsBSR2*/*CYP78A15*, *OsDSS1*/*CYP96B4*, *OsXyn1*/ *MORE1m*, *OsGW2*, *OsGW8*, and *OsKNAT7* (Ishimaru et al. 2013; Shim et al. 2022; Yin et al. 2020; Maeda et al. 2019; Tamiru et al. 2015; Hao et al. 2021; Xiao et al. 2018; Qi and Xiong 2013; Tu et al. 2020; Wang et al. 2012, 2015a, b; Liu et al. 2022; Chen et al. 2024). *OsZHD1* and *OsZHD2*, as transcript factor, regulate the gene expression. Then the loss-of-function of *OsZHD1* and *OsZHD2* whether can influence the expression level of the other grain size-related genes in the mutants? The results of RNA-seq data and qRT-PCR showed that *OsPUP7* and *OsKNAT7* were significantly down-regulated in the *oszhd1oszhd2* than ZH11 and single mutants (Fig. 8A and B; Table S7). Additionally, the other grain size-related genes were significantly down-regulated in the single and double mutants than the wild type. These results suggested *OsZHD1* and *OsZHD2* were required for the grain size development.

Tan and Irish (2006) showed ZHD TFs putatively bind to TAATTA *cis*-element, whether it obtains the binding sites in the promoter regions of cell cycle and expansion-related and grain size-related genes depicted in Fig. 8? The results displayed that 80% of these genes contained ZHD binding sites within their promoters (examining the ATG upstream 2000 bp region) (Fig. S6; Table S10). It is worth noting that the promoters of *OsEXPA19*, *OsCYCP4;3*, *OsTGW6*, *OsDSS1*, and *OsKNAT7* each contain three or more binding sites (Fig. S6; Table S10). In summary, the qRT-PCR analysis of these genes confirmed the high spatiotemporal resolution of our gene profiling dataset, and *OsZHD1*/*2* may bind to the promoters of these genes to exert regulatory functions, which will facilitate future investigation of the molecular mechanisms underlying *OsZHD1/2* with these genes in regulating grain size.

## Discussion

We herein identified that the loss-of-function of *OsZHD1* and *OsZHD2* were responsible for the mutants phenotype in grain size. The spikelet hull of *oszhd1oszhd2* was smaller than the wild type, *oszhd1-1*, and *oszhd2-1*. Through the histological observation and SEM analysis, the hull cross-section of the spikelet in *oszhd1oszhd2* revealed a significant decrease in total cell length and number, and single cell width in outer parenchyma compared with wild type, *oszhd1-1*, and *oszhd2-1*, respectively (Fig. 4B, C, and D). Furthermore, the results of scanning electron microscopy showed that the average length of longitudinal epidermal cells were increased, but the width were decreased in outer and inner glumes of *oszhd1oszhd2* than wild-type, *oszhd1-1*, *oszhd2-1*, respectively (Fig. 4E, F, G, and H). The overexpression of *OsZHD1* caused abaxial leaf curling and drooping leaf observed in *ACL-D* mutant, because of the abnormal number and the arrangement of bulliform cells (Xu et al. 2014). In *ACL-D* plants, the reduction of the number of cells caused the shorter length of internodes and the defective cell elongation induced shorter panicles. Overexpression of *OsZHD2*, the closest homolog *OsZHD1*, reappeared the abaxial curling leaf, and the expression level of *CYCD4;1* increased in the lateral roots of *OsZHD2-D* (*OsZHD2* overexpression mutant), suggesting that *OsZHD2* promotes cell cycle progression during the root growth. Interestingly, unlike the rolled-leaf phenotype reported in *ACL-D* mutant, overexpressed *AtZF-HD1* gene (the ortholog of *OsZHD1*) in *Arabidopsis* was observed normal leaf in all representative lines. This results indicated that the *ZHDs* control the leaf curling through affecting the bulliform cells that specifically existed in monocots rather than dicots plants (Xu et al. 2014). It is noteworthy that the expression level of cell cycle and expansion-related genes were down-regulated in the *oszhd1oszhd2*, suggesting that the decreased cell number and morphology might have resulted from the depressed expression of genes suppressing cell proliferation (Fig. 8A and B; Table S7).

In plants, the ZHD protein family displays an important diversity in terms of number and sequences across the plant kingdom (Hu et al. 2008). Multiple ZHD proteins are involved in meaningful biological processes such as vegetative and reproductive development (Bollier et al. 2022). *ZHD5* was one of the first reported *ZHD* TF acting on plant development in *Arabidopsis* (Tan and Irish 2006). The *ZHD5* overexpressing lines display accelerated growth with larger leaves containing larger cells, altered floral architecture, and promoted shoot regeneration by regulating cytokinin response (Seo et al. 2011). The expression level of *GA3OX2* (specifically expressed in germinating seeds and hypocotyls) is increased in the *ZHD1*-overexpressing lines (*athb25-1D*), resulting in the more GA bioactive accumulation than wild-type plants and in larger hypocotyl cells, bigger seeds, and seed longevity (Bueso et al. 2014, 2016). Moreover, seed longevity was even more reduced not only in *ZHD1*, but also in *ZHD2* and *ZHD4* transgenic lines using antisense strategy, indicating the occurrence of a functional redundancy in GA signaling among *ZHD* genes (Bueso et al. 2014).

The overexpression of *OsZHD1* or *OsZHD2* leads to dwarfism and abaxial leaf curling in rice (Bollier et al. 2022). Additionally, overexpression of *OsZHD2* improves root growth by enhancing meristem activity and elevates ethylene concentrations by increasing the transcript levels ethylene biosynthesis genes in the *OsZHD2-D* mutant (Yoon et al. 2020). The volume of root system and overall yield were increased, especially under the poor nutritional status, and our results show loss-of-function of *OsZHD1* and *OsZHD2* induced the smaller grain size (Fig. 3; Fig. S4; Fig. S5), suggesting *OsZHD1* and *OsZHD2* are key traits that could be applied in the regulating of grain yield. The development of lateral roots were significantly diminished in the *OsZHD1* and *OsZHD2* double mutants rather than the single mutants, and decreased the transcript levels of *OsSAM*, *OsACS5*, *OsACO2*, *OsOASA2*, *OsTAR2*, and *OsYUCCA7* in the double mutants, suggesting that functional redundant is existed in the *OsZHDs* genes, and these genes are involved in the control of the biosynthesis of ethylene and auxin (Yoon et al. 2020).

*ZHDs* genes, as transcription factor, were shown to regulate numerous genes by direct binding to their promoter, because of no activation domain in most *ZHDs*, indicating that it is required to interact with other factors to trigger transcriptional activation (Tan and Irish 2006). The transcriptome and ChIP-seq experiments showing that *ZHD* genes are the targets of the floral homeotic TFs, such as *AG*, *AP3*, *PI*, and *SEP3* (Perrella et al. 2018). The protein-protein interaction studies revealed that ZHD3, ZHD8, and ZHD11 interact with the stress responsive protein HIPP (HEAVY METAL ASSOCIATED ISOPRENYLATED PLANT PROTEIN)(Barth et al. 2009). ZHD11 could interact with NAC TFs obtaining an enriched His domain, which could be participated in metal ion binding (Tran et al. 2007). The *ARF2* (*AUXIN RESPONSE FACTOR 2*) directly represses the expression of *ZHD5* through binding the AuxRE (auxin response elements) located in the *ZHD5* promoter to response drought and ABA treatment (Bollier et al. 2022). In rice, OsZHD1, OsZHD2, OsZHD4, and OsZHD8 bind to the promoter sequence of *OsDREB1b*, which is a major regulator to response abiotic stresses, and all four are direct repressor of *OsDREB1b* (Figueiredo et al. 2012). In soybean, *GmZF-HD1* and *GmZF-HD2* were induced by pathogen infection, and they could bind to the promoter sequence to activate *GmCaM4* to increase resistance through the hypersensitive response-associated lesions (Bollier et al. 2022; Shi et al. 2021). In this study, we demonstrated that OsZHD1 could directly interact with OsZHD2, and both genes redundantly regulate the development of grain size by affecting the cell proliferation. Then *ZHD* genes should be considered as valuable targets in breeding and crop improvement programs.

## Conclusion

*ZHDs* obviously act in a numerous number of processes all across plant life. They act as vital transcriptional regulators to direct binding DNA through the homeodomain. ZHDs protein could interact not only with other family members but also with other protein partners from different families to regulate a large number of targeted genes. However, the molecular mechanisms and the genetic regulatory network underlying the function of ZHDs proteins remain almost undeciphered.

## Electronic Supplementary Material

Below is the link to the electronic supplementary material.

Supplementary Material 1

Supplementary Material 2

Supplementary Material 3

Supplementary Material 4

Supplementary Material 5

Supplementary Material 6

Supplementary Material 7

Supplementary Material 8

## Data Availability

All relevant data are provided in Supplementary Tables. The RNA-seq data for this study can be found in the National Center for Biotechnology Information (NCBI) Sequence Read Archive under the accession number PRJNA1203770.

## Declarations

### Accession Number

Sequence data in this study can be found in the Rice Genome Annotation Project data libraries under the following accession numbers: *OsZHD1* (LOC_Os09g29130); *OsZHD2* (LOC_Os08g37400); *OsEXPA13* (LOC_Os02g16730); *OsEXPA19* (LOC_Os03g06050); *OsEXPB3* (LOC_Os10g40720); *OsCYCP4;3* (LOC_Os02g03294); *OsCycF1;3* (LOC_Os02g39240); *OsCycF2;1* (LOC_Os02g39420); *OsCycF2;2* (LOC_Os02g39260); *OsCycT1;2* (LOC_Os02g24190); *OsTGW6* (LOC_Os06g41850); *OsLFS* (LOC_Os08g34360); *OsPUP4* (LOC_Os01g48800); *OsPUP6* (LOC_Os04g49748); *OsPUP7* (LOC_Os05g48300); *OsPUP10* (LOC_Os04g49739); *OsBSR2* (LOC_Os08g43390); *OsDSS1* (LOC_Os03g04680); *OsXyn1* (LOC_Os03g47010); *OsGW2* (LOC_Os02g14720); *OsGW8* (LOC_Os08g41940); *OsKNAT7* (LOC_Os03g03164).

## Abbreviations

DEGs: Differentially expressed genes
TF: Transcription factors
CRISPR: Clustered regularly interspaced short palindromic repeats
FAA: Formalin-Aceto-Alcohol
SEM: Scanning electron microscope
GUS: β-glucuronidase
Y2H: Yeast two-hybrid
BiFC: Bimolecular fluorescence complementation
YFP: Yellow fluorescent protein
Co-IP: Co-immunoprecipitation
GFP: Green fluorescent protein
RNA-seq: RNA sequencing
ChIP-seq: Chromatin immunoprecipitation sequencing
GO: Gene ontology
KEGG: Kyoto encyclopedia of genes and genomes
