# Supplementary figures and images for: The OsZHD1 and OsZHD2, Two Zinc Finger Homeobox Transcription Factor, Redundantly Control Grain Size by Influencing Cell Proliferation in Rice
Source: Rice (N Y). 2025 Mar 22;18:20. doi: 10.1186/s12284-025-00774-8 (PMC11928714; doi:10.1186/s12284-025-00774-8)

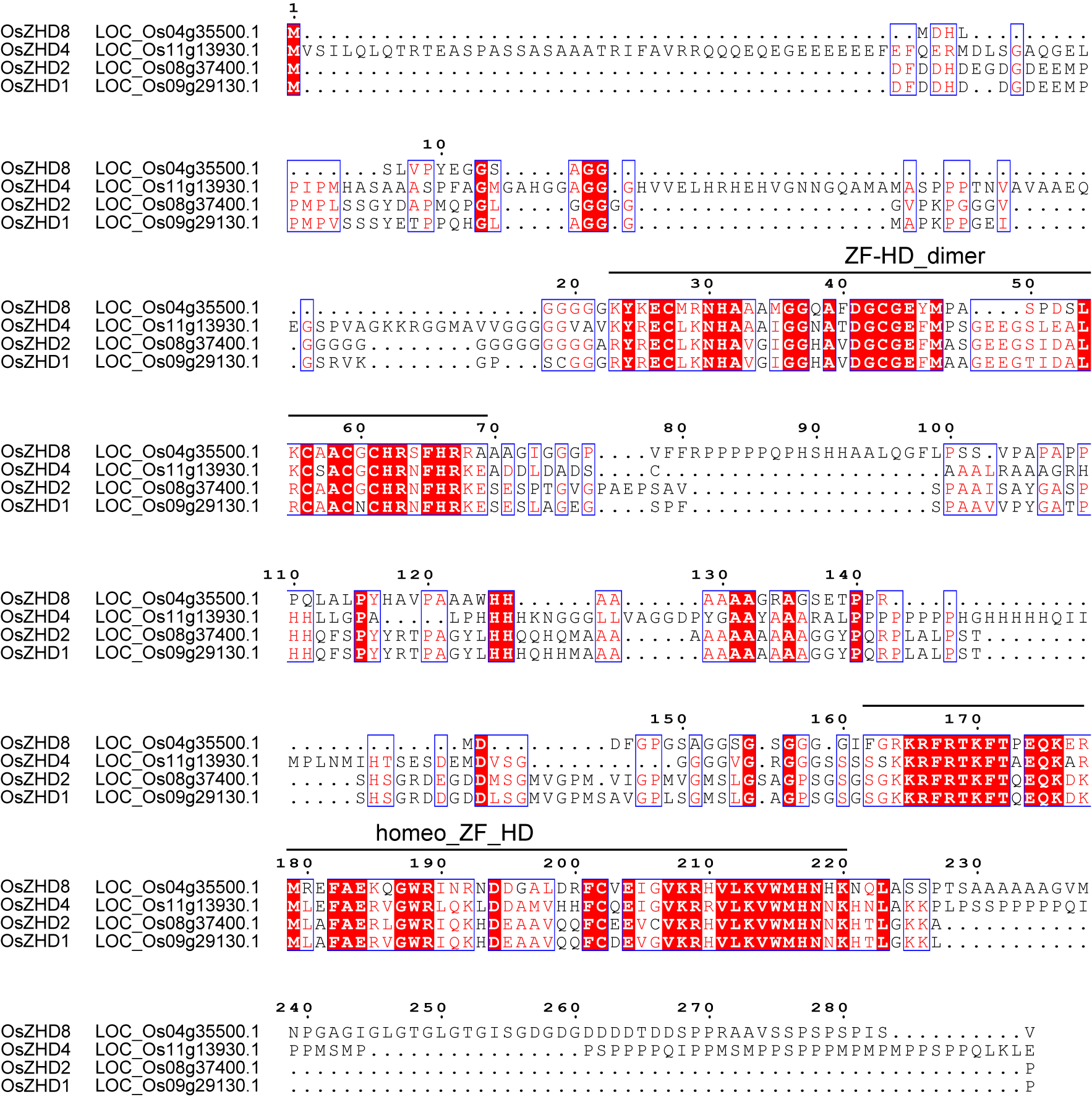

Supplement: Supplementary file 1 — Supplementary Material 1 [file 12284_2025_774_MOESM1_ESM.tif]

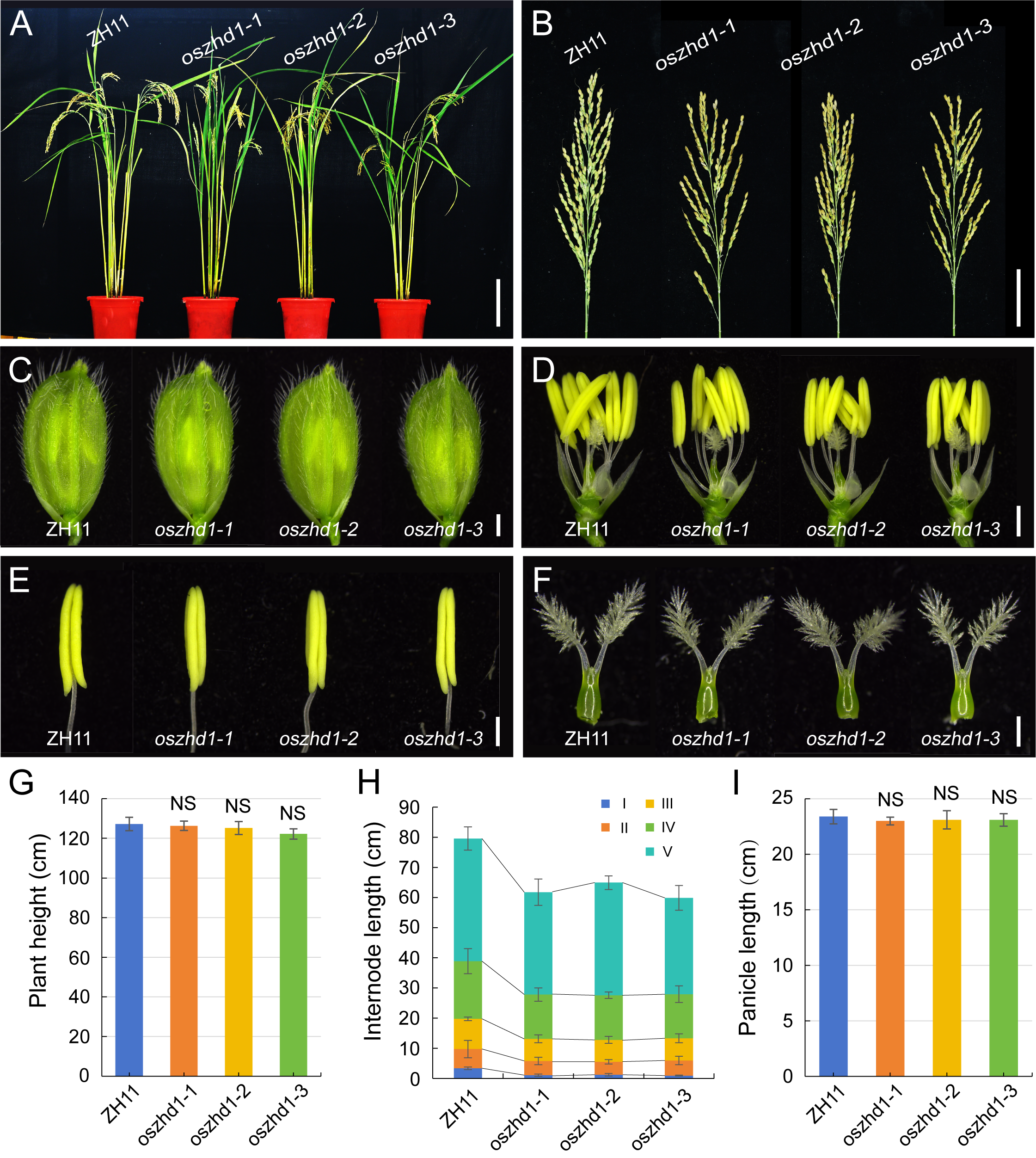

Supplement: Supplementary file 2 — Supplementary Material 2 [file 12284_2025_774_MOESM2_ESM.tif]

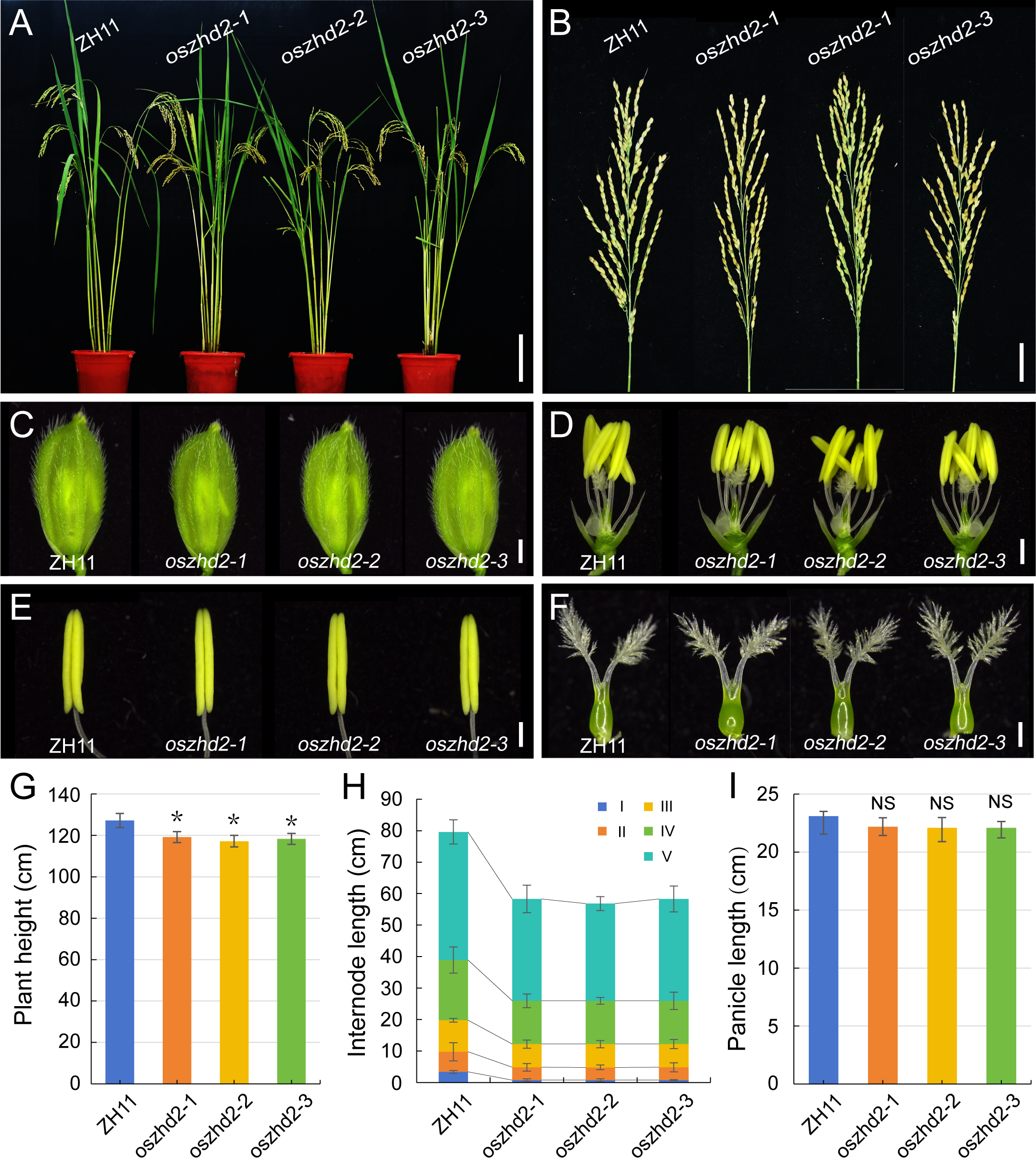

Supplement: Supplementary file 3 — Supplementary Material 3 [file 12284_2025_774_MOESM3_ESM.tif]

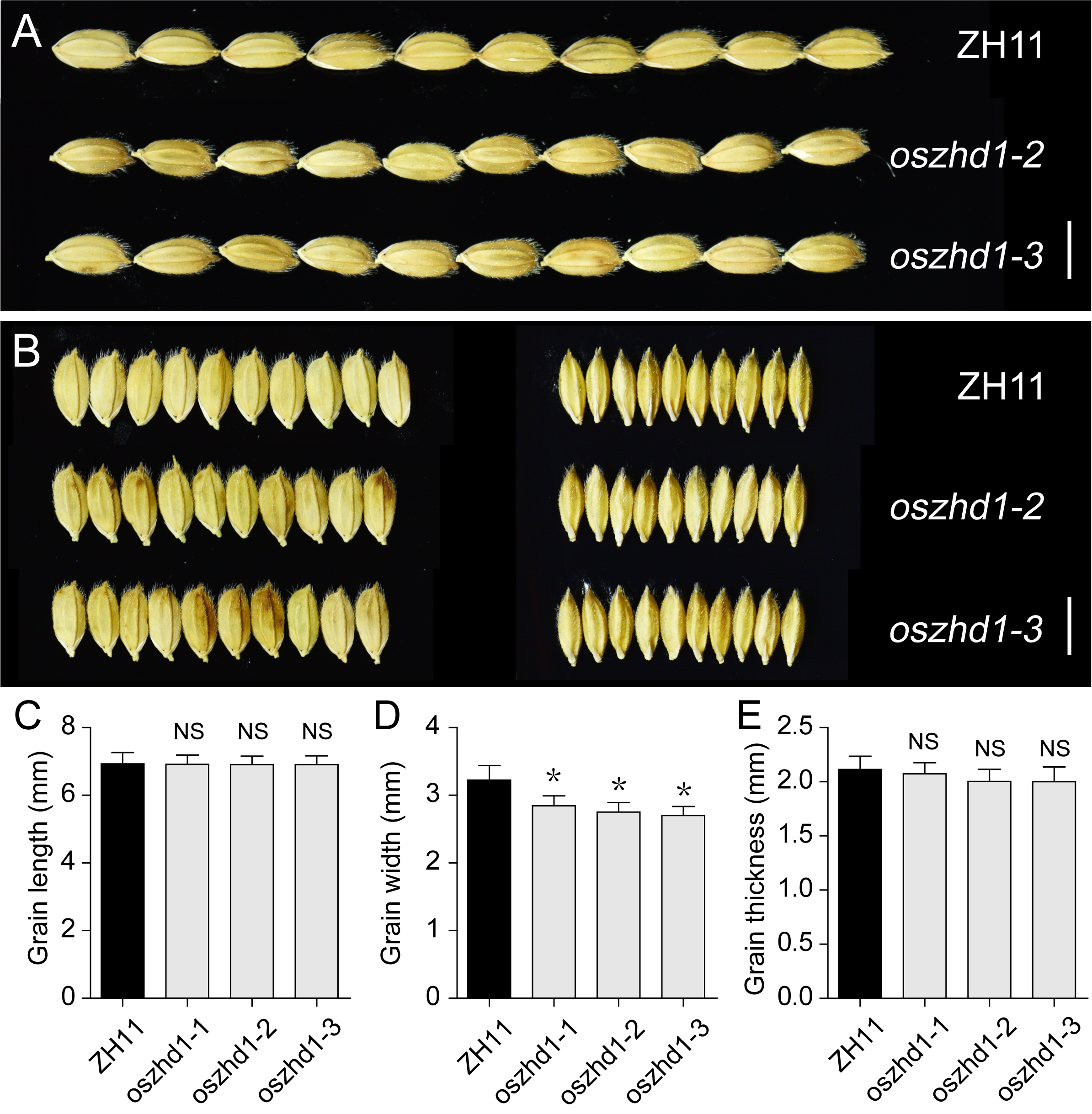

Supplement: Supplementary file 4 — Supplementary Material 4 [file 12284_2025_774_MOESM4_ESM.tif]

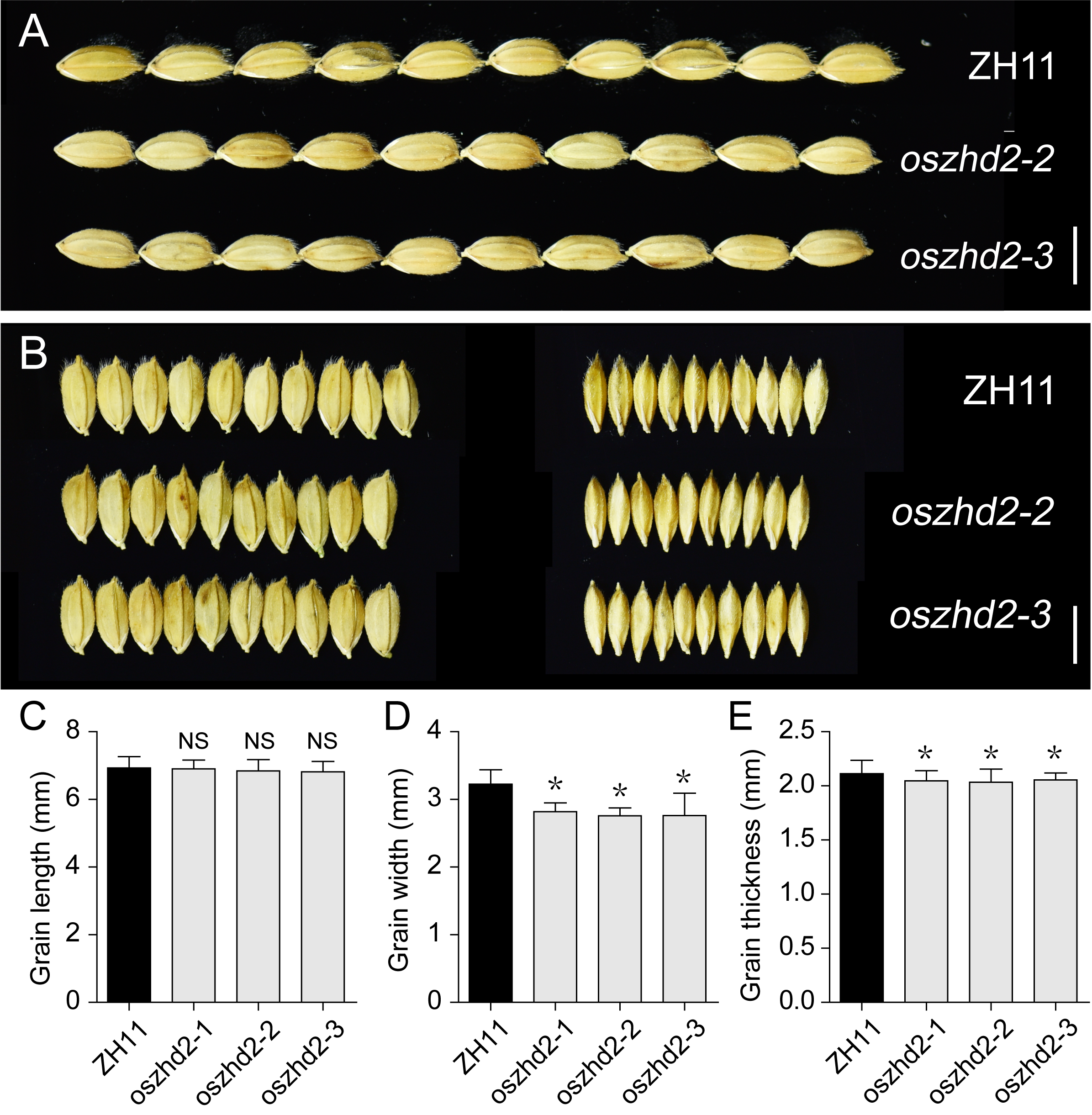

Supplement: Supplementary file 5 — Supplementary Material 5 [file 12284_2025_774_MOESM5_ESM.tif]

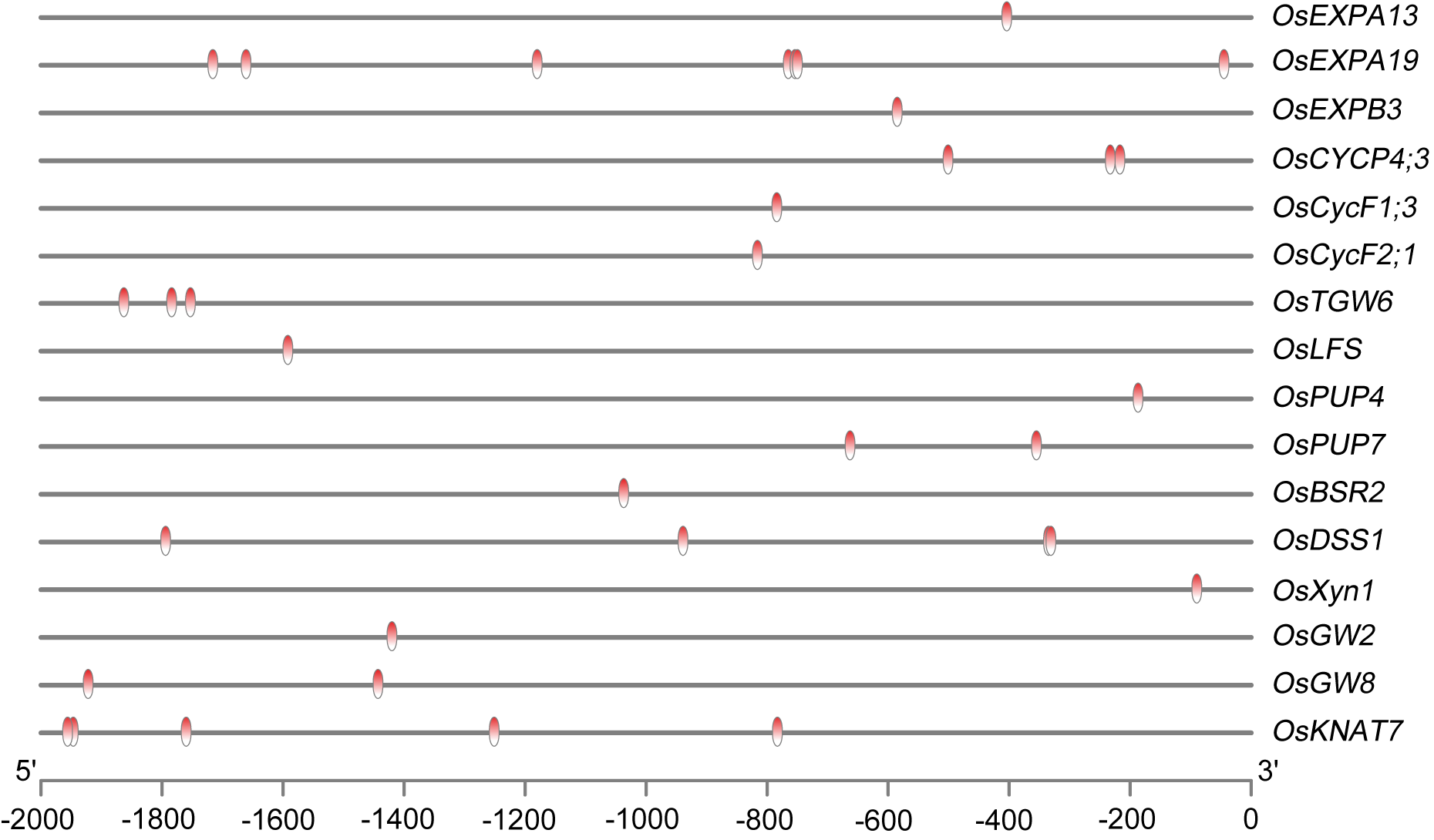

Supplement: Supplementary file 6 — Supplementary Material 6 [file 12284_2025_774_MOESM6_ESM.tif]
