# Supplementary material for: The OsZHD1 and OsZHD2, Two Zinc Finger Homeobox Transcription Factor, Redundantly Control Grain Size by Influencing Cell Proliferation in Rice
Source: Rice (N Y). 2025 Mar 22;18:20. doi: 10.1186/s12284-025-00774-8 (PMC11928714; doi:10.1186/s12284-025-00774-8)
Supplement: Supplementary file 8 — Supplementary Material 8 [file 12284_2025_774_MOESM8_ESM.docx]

**Supplementary Information**

**Figure S1 Amino acid sequence alignment of OsZHD1, OsZHD2, OsZHD4, and OsZHD8.**

Multiple sequence alignment of OsZHD1/2/4/8 proteins containing ZF-HD dimer and homeo_ZF_HD domains Conserved residues are highlighted in red, and specific residues are boxed in blue. The ZF-HD dimer and homeo_ZF_HD domains are indicated with horizontal lines.

ZF-HD_dimer: Zinc Finger Homeodomain Dimerization; homeo_ZF_HD: Homeobox Zinc Finger Homeodomain.

**Figure S2 Morphology analysis of the wild-type (ZH11) and *oszhd1.***

(A) The phenotype of wild-type (left, ZH11), *oszhd1-1*, *oszhd1-2*, *and oszhd1-3* plants at maturity. Bar=15cm.

(B) The panicles of ZH11, *oszhd1-1*, *oszhd1-2*, *and oszhd1-3.* Bar=5cm.

(C-D) The spikelets of ZH11, *oszhd1-1*, *oszhd1-2*, *and oszhd1-3*. Bar=1mm.

(E-F) Anthers and pistils of ZH11, *oszhd1-1*, *oszhd1-2*, *and oszhd1-3*. Bar=500μm.

(G-I) The average plant height, internode length, panicle length of ZH11, *oszhd1-1*, *oszhd1-2*, *and oszhd1-3*, respectively. (n=20 per sample).

Data are given as means ±SD. Different letters denote significant difference at P < 0.05 according to ANOVA in combination with Duncan’s multiple range test.

**Figure S3 Morphology analysis of the wild-type (ZH11) and *oszhd2.***

(A) The phenotype of wild-type (left, ZH11), *oszhd2-1*, *oszhd2-2*, *and oszhd2-3* plants at maturity. Bar=15cm.

(B) The panicles of ZH11, *oszhd2-1*, *oszhd2-2*, *and oszhd2-3.* Bar=5cm.

(C-D) The spikelets of ZH11, *oszhd2-1*, *oszhd2-2*, *and oszhd2-3*. Bar=1mm.

(E-F) Anthers and pistils of ZH11, *oszhd2-1*, *oszhd2-2*, *and oszhd2-3*. Bar=500μm.

(G-I) The average plant height, internode length, panicle length of ZH11, *oszhd2-1*, *oszhd2-2*, *and oszhd2-3*, respectively. (n=20 per sample).

Data are given as means ±SD. Different letters denote significant difference at P < 0.05 according to ANOVA in combination with Duncan’s multiple range test.

**Figure S4 Functional verification of *OsZHD1* in grain size.**

(A-B) Comparation on grain size of *oszhd1-2* and *oszhd1-3* with ZH11, respectively. Bar=5mm.

(C-E) Statistical analysis of grain length, width, and thickness of ZH11, *oszhd1-1*, *oszhd1-2*, *and oszhd1-3*. (n=50 per sample).

Data are given as means ±SD. Different letters denote significant difference at P < 0.05 according to ANOVA in combination with Duncan’s multiple range test.

**Figure S5 Functional verification of *OsZHD2* in grain size.**

(A-B) Comparation on grain size of *oszhd2-2* and *oszhd2-3* with ZH11, respectively. Bar=5mm.

(C-E) Statistical analysis of Grain length, width, and thickness of ZH11, *oszhd2-1*, *oszhd2-2*, *and oszhd2-3*. (n=50 per sample).

Data are given as means ±SD. Different letters denote significant difference at P < 0.05 according to ANOVA in combination with Duncan’s multiple range test.

**Figure S6 Putative ZF-HD TF binding sites in the promoter regions of cell cycle and expansion-related and grain size-related genes.**

Quantity and position of ZF-HD binding element (TAATTA) detected in the promoter regions (sequence retrieved from about ≤ 2 kb upstream region) of cell cycle and expansion-related and grain size-related genes via PlantCARE analysis.
